# Music expertise differentially modulates the hemispheric lateralization of music reading

**DOI:** 10.3389/fcogn.2024.1403584

**Published:** 2024-08-19

**Authors:** Sara Tze Kwan Li

**Affiliations:** Department of Social Sciences, School of Arts and Social Sciences, Hong Kong Metropolitan University, Hong Kong, China

**Keywords:** hemispheric lateralization, music expertise, music reading, musical elements, brain plasticity

## Abstract

Previous studies have shown that music expertise relates to the hemispheric lateralization of music reading among musicians and non-musicians. However, it remains unclear that how music expertise modulates the hemispheric lateralization of music reading along the music learning trajectory and how music expertise modulates the hemispheric lateralization of reading different musical elements. This study examined how music expertise modulates the hemispheric lateralization of music reading in pitch elements (e.g., pitch, harmony), temporal elements (e.g., rhythm), and expressive elements (e.g., articulation) among musicians, music learners, and non-musicians. Musicians (*n* = 38), music learners (*n* = 26), and non-musicians (*n* = 33) worked on a set of divided visual field sequential matching tasks with four musical elements, i.e., pitch, harmony, rhythm, and articulation, in separate blocks. An eye-tracker was used to ensure participants' central fixation before each trial. Participants judged whether the first and second target stimuli were the same as quickly and accurately as possible. The findings showed that for musicians, no significant differences were observed between the left visual field (LVF) and the right visual field (RVF), suggesting musicians' bilateral representation in music reading. Music learners had an RVF/LH (left hemisphere) advantage over the LVF/RH (right hemisphere), suggesting music learners tended to be more left-lateralized in music reading. In contrast, non-musicians had an LVF/RH advantage over the RVF/LH, suggesting non-musicians tended to be more right-lateralized in music reading. In addition, music expertise correlates with the laterality index (LI) in music reading, suggesting that the better the overall performance in music expertise task, the greater the tendency to be more left-lateralized in music reading. Nonetheless, musicians, music learners, and non-musicians did not show different visual field effects in any individual musical elements respectively, suggesting the cognitive processes involved might share similar lateralization effects among the three groups when only one particular musical element is examined. In general, this study suggests the effect of music training on brain plasticity along the music learning trajectory. It also highlights the possibilities that bilateral or left hemispheric lateralization may serve as an expertise marker for musical reading.

## 1 Introduction

Music reading is a complex cognitive process. It involves visual perception and encoding of multiple musical elements from a single representation of musical notation in Western music. Its complexity served as an excellent example to examine how human mind processes multiple visual elements simultaneously. For example, a single note, crotchet A_4_ with a staccato mark, represents its pitch (i.e., A_4_, a pitch with a frequency of 440 Hz), rhythm (i.e., crotchet; a musical note with a time value of a quarter of a semibreve/whole note), and articulation (i.e., staccato; a detached note, as a musical expression). In addition, a C major triad, represents its harmony (i.e., a C major chord with three notes, pitches C-E-G, indicating the root, major third, and perfect fifth of C major). The two examples above highlighted important musical elements in music reading, including pitch elements (e.g., pitch, harmony), temporal elements (e.g., rhythm), and expressive elements (e.g., articulation).

In music reading, pitch is defined as the spatial location of a musical notation on the staff with five lines and four spaces, representing low to high pitches based on the rate of vibration per second (i.e., frequency). Harmony refers to the simultaneous presentation of two or more musical notations on the staff, forming chords or triads, which usually involves three notes indicating the root, third, and fifth note of a key. Rhythm refers to the temporal aspects of music on the staff, including the grouping of notes into beats, and grouping of beats into bars (i.e., specific measures indicating metrical divisions of music; Kennedy et al., 2013). Articulation refers to the musical expressive markings on the staff, indicating an act of sound making (Articulation, 2024). Legato (i.e., smooth) and staccato (i.e., detached) are common articulatory expressive markings in musical scores (Kennedy et al., 2013). The four musical elements, i.e., pitch, harmony, rhythm, and articulation, highlighted the multifaceted nature of music reading.

With such complexity, musicians, i.e., who receive extensive music training to read music regularly, remember and integrate visual and auditory musical information (see Zhang et al., 2020), have shown superior music reading performance when compared with non-musicians, i.e., who do not play or read music or those with very minimal exposure of music. Previous studies have characterized music reading expertise using different expertise markers, such as behavioral responses indicated by a higher accuracy (ACC) and faster response time (RT) in music reading tasks. For instance, musicians had a significantly higher ACC and about 150 ms faster RT than non-musicians in a musical note recognition task (Proverbio et al., 2013). In addition, musicians were 550 ms faster than non-musicians in a four-note musical sequence matching task (Wong and Gauthier, 2010).

On the other hand, music reading expertise has been possibly characterized by another expertise marker, i.e., hemispheric lateralization, among musicians and non-musicians in music reading. Hemispheric lateralization refers to the cerebral dominance between the left hemisphere (LH) and the right hemisphere (RH) during cognitive processes (Hellige, 1990). Recent studies examining hemispheric laterization in music reading usually employed neuroimaging techniques, e.g., electroencephalogram (EEG)/event-related potentials (ERPs; e.g., Pantaleo et al., 2024), or functional magnetic resonance imaging (fMRI; e.g., Wong and Gauthier, 2010). The measurements indicated a higher amplitude in some specific electrodes or stronger activation in some localized brain areas either in the LH, RH, or both hemispheres, and further suggested the hemispheric lateralization of the cognitive processes involved during music reading.

Consistent findings had shown that musicians had a bilateral processing of music reading, while non-musicians tended to be more right-lateralized in music reading. For instance, a recent study by Pantaleo et al. (2024) had shown that musicians had a bilateral N170 responses to musical notes in a note detection task with high density EEG/ERPs measured. A follow-up analysis using standardized weighted low-resolution electromagnetic tomography (swLORETA) source reconstructions had further revealed that musicians' right middle occipital gyrus (MOG BA37) and the left middle occipital gyrus (MOG BA19) were active during note processing, while for non-musicians, only right precuneus BA7 was active.

Similar findings were shown in other previous neuropsychological studies. In an EEG/ERPs-swLORETA study by Proverbio et al. (2013), musicians had a bilateral processing of musical notes, indicating by the involvement of left fusiform gyrus (BA37) and the right fusiform gyrus (BA38) in a note recognition task. In contrast, non-musicians had a right-lateralized processing of musical notes, indicating by the involvement of the right visual areas (BA19). Additionally, in an fMRI study by Wong and Gauthier (2010), musicians had shown higher neural responses in the bilateral fusiform gyrus and the bilateral early visual areas (V1/V2) than non-musicians in a gap detection task with musical notes presented. Such differences observed in hemispheric lateralization among musicians and non-musicians in music reading further suggested the effect of music training on brain plasticity, i.e., the brain's ability to change its structures and functions due to extensive experiences (Kolb and Whishaw, 1998; Rodrigues et al., 2010).

It is debatable that how different musical elements were processed regarding their perceptual characteristics. Most studies focused on examining the hemispheric lateralization effects of visual processing of pitch elements. In pitch reading, a bilateral processing were observed among musicians and non-musicians. For example, in a magnetoencephalography (MEG) study by Lu et al. (2019), musicians showed bilateral activations at the superior parietal cortex during one-musical-note pitch reading task and five-musical-note pitch reading task. Additionally, a recent EEG/ERP study by Proverbio et al. (2024) showed that, participants, including musicians and non-musicians (with about 3 years of music study during high school), had bilateral N170 responses in musical note selection based on pitch judgment, in which the responses may potentially be larger over the right hemisphere. Similarly, Li and Hsiao (2018) showed that no different hemispheric lateralization effects were observed between musicians and non-musicians in a pitch sequential matching task, suggesting the bilateral processing of pitch for both groups. The bilateral processing of pitch may potentially be explained by the global and local information processed in reading. Music readers needed to attend to the five-line staff as global information, as well as the specific note locations as local information. The right-lateralized global processing and left-lateralized local processing (Fink et al., 1997) may contribute to the bilateral processing of pitch reading.

In chord reading, a left-lateralized or bilateral processing advantage was observed among musicians. In Segalowitz et al. (1979), musicians or amateur musicians had shown a left-lateralized processing advantage in a divided visual field chord playing task. A similar left-lateralized processing advantage was also observed in Salis (1980). However, Li and Hsiao (2018) showed that no different hemispheric lateralization effects were observed between musicians and non-musicians in a chord sequential matching task, suggesting the bilateral processing of harmony for both groups, which may also be explained by the processing of global information of the five-line staff and local information as the specific note locations in chord reading. Here the discrepancies of the lateralization effects in chord reading observed in different studies may possibly be explained by the task nature. For example, Segalowitz et al. (1979) and Salis (1980) involved chord playing in tasks, while Li and Hsiao (2018) required a sequential matching of chords without playing. Since chord playing involves motor planning, which may induce more left hemispheric lateralization effect (Janssen et al., 2011).

The above findings might have indicated that pitch and chord reading might have a similar bilateral processing mechanisms in reading. Nonetheless, limited studies were conducted to further investigate the hemispheric lateralization of other musical elements in reading. Additionally, the above studies were conducted using different tasks and methodologies, making a direct comparison of the hemispheric lateralization effects among different musical elements difficult.

In the literature, Ono et al. (2011) has served as a close example examining the hemispheric lateralization effects of different musical elements in the auditory domain. This study examined how music expertise influences hemispheric lateralization of auditory musical elements, including pitch, chord, rhythm and timbre using MEG. In general, musicians showed a bilateral activations, while non-musicians showed a right-lateralized advantage in auditory oddball tasks with the four musical elements. However, no specific hemispheric lateralization effects were observed respectively among pitch, chord, rhythm, and timbre, between musicians and non-musicians. This study showed that music expertise generally modulates hemispheric lateralization of auditory music processing.

This line of research demonstrates how extensive training of music may possibly be related to the auditory processing mechanisms of the two hemispheres and expand our understanding of the effects on brain plasticity. To further expand the scope of study to the visual domain, examining how music expertise influences hemispheric lateralization of music reading will provide substantial information. Based on Ono et al. (2011), musical elements, such as pitch, chord, and rhythm, are commonly shared between the visual and auditory domains. In contrast, timbre (i.e., tone color; Kennedy et al., 2013) is an auditory musical element without any explicit representations on musical notations. Thus, the current study aims to include pitch, chord, and rhythm as three out of the four musical elements examined. A systematic review and meta-analysis of 30 fMRI studies indicated a bilateral auditory processing of musical rhythms (Kasdan et al., 2022). Furthermore, in a fMRI and MEG study, a preferential processing has been shown in the left hemisphere for relatively faster rhythms, and in the right hemisphere for relatively slower rhythms (Pflug et al., 2019), indicating the involvement of both hemispheres in rhythmic auditory processing. In addition, the current study also aims to include articulation as one of the musical elements examined. However, no or very limited studies had examined the processing of articulatory markings (e.g., slurs, staccatos) in music reading. In this study, the term “music reading” refers to the process of making visual judgment of pitch elements (e.g., pitch, harmony), temporal elements (e.g., rhythm), and expressive elements (e.g., articulation) based on musical scores (i.e., five-line staff) as the visual input.

In terms of the levels of music expertise, most previous studies recruited musicians and non-musicians to examine how music expertise influences hemispheric lateralization using a quasi-experimental design (e.g., Wong and Gauthier, 2010; Proverbio et al., 2013, 2024; Li and Hsiao, 2018). While the possibly different hemispheric lateralization effects observed between musicians and non-musicians could be attributed by their differential music expertise, it remains unclear how did the change of hemispheric lateralization happen along the music learning trajectory. Thus, it is crucial that the study participants includes music learners (i.e., participants with some formal music training experience and music exposure) in addition to musicians and non-musicians. This will facilitate our understanding of how music expertise influences hemispheric lateralization throughout the music learning trajectory.

In general, this study aims to examine how music expertise modulates the hemispheric lateralization of music reading, and explore the effect of music training on brain plasticity along the music learning trajectory using a quasi-experimental design. More specifically, this study provides an overview of whether and how music expertise modulates the hemispheric lateralization of music reading of pitch elements (e.g., pitch, harmony), temporal elements (e.g., rhythm), and expressive elements (e.g., articulation).

Here we recruited musicians, music learners, and non-musicians for a set of divided visual field sequential matching tasks involving pitch elements (e.g., pitch, harmony), temporal elements (e.g., rhythm), and expressive elements (e.g., articulation) in separate blocks. The task was designed based on the contralateral relationships between the visual fields (VFs) and hemispheres, as represented by the right visual field-left hemisphere (RVF/LH) and left visual field-right hemisphere (LVF/RH) (Bourne, 2006), as well as the recommendations outlined in Hunter and Brysbaert (2008). Participants' central fixation at the beginning of each trial was monitored using an eye-tracker.

For each trial, participants judged whether the first target stimulus presented either at the left visual field (LVF) or the right visual field (RVF), as indicated by a central arrow, was the same as the second target stimulus presented at the center. The same trials referred to trials with identical first and second target stimuli, while different trials referred to the first and second target stimuli differed in terms of pitch/harmony/rhythm/articulation. Each musical element was presented separately in the respective block.

Based on the findings from previous studies examining hemispheric lateralization of music reading (e.g., Segalowitz et al., 1979; Salis, 1980; Wong and Gauthier, 2010; Proverbio et al., 2013; Mongelli et al., 2017; Li and Hsiao, 2018; Pantaleo et al., 2024), musicians were hypothesized to have a bilateral processing advantage in music reading. In contrast, non-musicians were hypothesized to have an LVF/RH advantage over the RVF/LH in music reading. These hypotheses were consistent with the findings from previous studies (e.g., Proverbio et al., 2013; Pantaleo et al., 2024), suggesting music expertise increases the tendency of left lateralization of music reading (Mongelli et al., 2017), and resulting in bilateral processing due to the extensive music reading experience. On the other hand, the hypothesis was also in-lined with the idea that music notations did not represent any specific meanings to non-musicians, non-musicians may employ different visuo-spatial processing skills to process musical notation. The visuo-spatial processing tended to be right-lateralized (e.g., Bever and Chiarello, 1974; Patston et al., 2006).

In addition, music learners were hypothesized to have an RVF/LH advantage over the LVF/RH in music reading. This hypothesis could possibly be supported by a finding from Wong and Gauthier (2010), showing that non-musicians who had 0.8 years of music reading experience had a higher activation in the left occipito-temporal junction when compared with musicians in a gap detection task in music scores. Furthermore, this hypothesis was also related to the possibility that music learners rely on analytic processing to focus on the details of musical notations, which tends to be left-lateralized (Bever and Chiarello, 1974), during music reading.

More specifically, given the findings from some attempts examining the hemispheric lateralization of musical elements in music reading, for pitch, musicians, music learners, and non-musicians were hypothesized to show a bilateral processing, as supported by previous studies (e.g., Li and Hsiao, 2018; Lu et al., 2019; Proverbio et al., 2024). While for harmony, it was hypothesized that musicians, music learners, and non-musicians had a left-lateralized or bilateral processing, as supported by previous studies (e.g., Segalowitz et al., 1979; Salis, 1980; Li and Hsiao, 2018). Both hypotheses could be explained by the right-lateralized global processing of five-line staff and left-lateralized local processing of specific note locations.

For rhythm and articulation, based on the limited previous studies, it remains unclear whether musicians, music learners, and non-musicians show different hemispheric lateralization when reading. Yet, based on their perceptual characteristics, for rhythm and articulation, musicians, music learners, and non-musicians were hypothesized to showed a bilateral processing regarding the right-lateralized global processing of the overall rhythmic and articulatory patterns, and left-lateralized local processing of specific rhythmic and expressive markings.

In short, this study explores how music expertise modulates the hemispheric lateralization of music reading among musicians, music learners, and non-musicians. It may potentially reveal different processing mechanisms in various musical elements, including pitch elements (e.g., pitch, harmony), temporal elements (e.g., rhythm), and expressive elements (e.g., articulation), along the music learning trajectory.

## 2 Methods

### 2.1 Participants

We recruited 97 participants from Hong Kong, aged 18–33, with a first language of Cantonese and an educational qualification of college or above. They had normal or corrected-to-normal vision, despite three reporting mild visual impairments (e.g., color deficiency) which did not affect their music reading abilities in the study. No participants reported any auditory impairments. The participants were classified into three groups according to their music training background, including musicians (*n* = 38), music learners (*n* = 26), and non-musicians (*n* = 33).

A priori power analysis with *F*-tests ANOVA: Repeated measures, within-between interaction, was conducted using G^*^Power version 3.1.9.6 for Mac OS X 10.7 to 14 (Faul et al., 2007) to examine the required minimum sample size for this study. The result revealed that, at least 60 participants (i.e., 20 participants per group) are needed to achieve 80% power for detecting a small effect at a significance criterion of α = 0.05. Thus, the recruited sample size of the musicians, music learners, and non-musicians fulfilled the suggested sample size indicated by G^*^Power.

The musicians (*n* = 38; 19 males, 19 females; *mean age* = 21, *range* = 18–32) were well-trained pianists/western instrumentalists who read music scores regularly. They had attained at least a Grade 8 or above in the Associated Board of the Royal Schools of Music (ABRSM) graded music examinations or equivalent in their primary instruments, including piano (*n* = 24), violin (*n* = 8), flute (*n* = 4), saxophone (*n* = 1), and clarinet (*n* = 1). They had started music learning between age 3 and 16 (*mean* = 5.92, *SD* = 2.59), and had 7–26 years of experience in music playing (*mean* = 14.26, *SD* = 3.74). Some musicians engaged in orchestral playing (*n* = 13), wind-band playing (*n* = 1), and choral conducting (*n* = 2).

The music learners (*n* = 26; 10 males, 16 females; *mean age* = 20.35, *range* = 18–33) were piano/western instrumental learners who read music scores regularly. They had attained Grades 2–5 in the Associated Board of the Royal Schools of Music (ABRSM) graded music examinations or equivalent in their primary instruments, including piano (*n* = 14), violin (*n* = 4), flute (*n* = 2), saxophone (*n* = 1), clarinet (*n* = 1), trumpet (*n* = 2), horn (*n* = 1), and guitar (*n* = 1). They had started music learning between age 3 and 16 (*mean* = 7.92, *SD* = 3.31), and had 1–18 years of experience in music playing (*mean* = 8.27, *SD* = 4.53). Some music learners engaged in orchestral playing (*n* = 5), wind-band playing (*n* = 1), and choir singing (*n* = 1).

The non-musicians (*n* = 33; 17 males, 16 females; *mean age* = 20.70, *range* = 18–27) were counterparts who had not received any formal music training in western instruments and did not read music scores, but matched in terms of demographics, handedness and working memory with the musicians and music learners.

Despite music training backgrounds, differential musical abilities among musicians, music learners, and non-musicians had been reflected in other measurements in this study. A set of music expertise tasks, comprising a visual memory and a visual-auditory music task, was included in this study. The visual memory task examined participants' ability to judge whether a bar has been shown in the previously shown four-bar, diatonic musical phrases (i.e., phrases made up of notes of a prevailing key) or non-diatonic musical phrases (i.e., phrases made up of chromatic notes that are out of a prevailing key), while the visual-auditory music task examined participants' ability to judge whether a visually shown musical phrase matched with an auditorily played musical phrase. The analysis of the music expertise task was based on the average accuracy (ACC) and response time (RT) of the visual memory task and visual-auditory music task. Musicians, music learners, and non-musicians were significantly different in ACC, with a large effect size [*F*_(2, 94)_ = 12.44, *p* < 0.001, ηp^2^ = 0.21], while no significant differences were observed in the RT [*F*_(2, 92)_ = 1.10, *p* = 0.33]. In ACC, *post-hoc* comparisons with Tukey HSD correction showed that musicians (*mean* = 67.68; *SD* = 10.43) significantly outperformed music learners (*mean* = 58.05; *SD* = 9.06) and non-musicians (*mean* = 58.43; *SD* = 7.22), while no significant differences were observed between music learners and non-musicians. Table 1 shows the participants' performance of the visual memory task and visual-auditory music tasks respectively.

**Table 1:** The accuracy (ACC) and correct response time (RT) of the music expertise tasks among musicians, music learners, and non-musicians.

	**Accuracy (ACC)**			**Correct response time (RT)**		
	***M***	***SD***	***Range***	***M***	***SD***	***Range***
**a. Music expertise task - visual memory task**						
Musicians	63.98	12.95	43.75–93.75	1,910.47	477.39	895.57–2797.85
Music learners	57.93	10.09	31.25–81.25	1,733.76	497.44	1,016.20–3053.75
Non-musicians	62.12	11.89	43.75–93.75	1,563.80	433.44	860.63–2,870.30
**b. Music expertise task - visual-auditory task**						
Musicians	71.38	16.86	0–100	834	335.04	236.63–1,592.3
Music learners	58.17	12.35	37.5–81.25	1028.01	477.22	327.4–2,181.6
Non-musicians	54.73	10.72	31.25–75	968.35	501.06	236.63–2,223.67

Similar differential musicality ratings among musicians, music learners, and non-musicians were also observed in the Goldsmiths Musical Sophistication Index (Gold-MSI; Müllensiefen et al., 2014). Gold-MSI is a self-reported questionnaire measuring musicality (i.e., multifaceted and broad musical skills and behaviors) among the general population with diverse musical background and expertise. Gold-MSI consisted of 38 items in five subscales, including (i) active engagement, representing active musical engagement behaviors, and the use of time and money on musical activities; (ii) perceptual abilities, representing cognitive musical abilities, and music listening skills; (iii) musical training, representing musical training and practice, and the degree of musicianship; (iv) singing abilities, representing singing skills and activities; and (v) emotions, representing emotional responses to music. The five factors contributed to a general musical sophistication factor, demonstrating the musicality among the general population (Müllensiefen et al., 2014). The participants indicated whether each of the statement described their music-related behaviors in a 7-point Likert scale, ranging from 1 (completely disagree) to 7 (completely agree). Using Gold-MSI in the current study allowed a more precise measurement of individual differences in musical skills and behaviors among musicians, music learners, and non-musicians.

As shown in the Gold-MSI (Müllensiefen et al., 2014), musicians, music learners, and non-musicians were significantly different in the sum scores of general musical sophistication, with a large effect size [*F*_(2, 94)_ = 65.57, *p* < 0.001, ηp^2^ = 0.58]. *Post-hoc* comparisons with Tukey HSD correction showed that musicians (*mean* = 87.21; *SD* = 9.41) significantly outperformed music learners (*mean* = 79.15; *SD* = 14.07) and non-musicians (*mean* = 55.67; *SD* = 12.45) in general musical sophistication, while music learners also significantly outperformed non-musicians. In addition, the three groups also significantly differed in the sum scores of other five subscales, with medium to large effect sizes, including active engagement [*F*_(2, 94)_ = 9.96, *p* < 0.001, ηp^2^ = 0.18], perceptual abilities [*F*_(2, 94)_ = 24.32, *p* < 0.001, ηp^2^ = 0.34], musical training [*F*_(2, 94)_ = 364.01, *p* < 0.001, ηp^2^ = 0.89], singing abilities [*F*_(2, 94)_ = 16.31, *p* < 0.001, ηp^2^ = 0.26], and emotions [*F*_(2, 94)_ = 4.93, *p* < 0.001, ηp^2^ = 0.10]. Table 2 shows the *post-hoc* comparisons with Tukey HSD correction, in which suggesting musicians outperformed non-musicians in all subscales, while music learners outperformed non-musicians in active engagement, perceptual abilities, musical training, and singing abilities. Musicians also outperformed music learners in perceptual abilities and musical training.

**Table 2:** *Post-hoc* comparisons with Tukey HSD correction of participants' musicality measured using the Goldsmiths Musical Sophistication Index (Gold-MSI).

					**95% CI**	
	**Comparisons**	**Mean differences (I-J)**	**Std. error**	**Sig**.	**Lower bound**	**Upper bound**
**a. Active engagement**						
Musicians	Music learners	1.53	1.82	0.68	−2.81	5.87
	Non-musicians	7.35	1.70	0.000^***^	3.29	11.41
Music learners	Non-musicians	5.82	1.88	0.007^**^	1.35	10.29
**b. Perceptual abilities**						
Musicians	Music learners	5.00	1.81	0.019^*^	0.68	9.32
	Non-musicians	11.82	1.70	0.000^***^	7.78	15.86
Music learners	Non-musicians	6.82	1.87	0.001^***^	2.37	11.27
**c. Musical training**						
Musicians	Music learners	5.56	0.99	0.000^***^	3.21	7.91
	Non-musicians	24.20	0.92	0.000^***^	22.00	26.40
Music learners	Non-musicians	18.64	1.02	0.000^***^	16.22	21.06
**d. Singing abilities**						
Musicians	Music learners	2.35	1.67	0.342	−1.63	6.34
	Non-musicians	8.75	1.56	0.000^***^	5.03	12.48
Music learners	Non-musicians	6.40	1.72	0.001^***^	2.30	10.50
**e. Emotions**						
Musicians	Music learners	0.374	1.14	0.94	−2.34	3.09
	Non-musicians	3.15	1.07	0.11^*^	0.61	5.69
Music learners	Non-musicians	2.78	1.18	0.52	−0.02	5.57
**f. General musical sophistication**						
Musicians	Music learners	8.06	3.02	0.024^*^	0.88	15.24
	Non-musicians	31.54	2.82	0.000^***^	24.83	38.26
Music learners	Non-musicians	23.49	3.11	0.000^***^	16.09	30.88

^*^p < 0.05, ^**^p < 0.01, ^***^p < 0.001.

A similar finding was observed among the three groups in a self-report familiarity rating of a musical note (i.e., a D5 crotchet). The participants indicated their familiarity toward the note based on a 10-point Likert scale (i.e., 1 represents having no knowledge in that musical note at all, and 10 represents being extremely familiar to that musical note). A significant difference was found in the familiarity rating of the musical note among musicians, music learners, and non-musicians, with a large effect size [*F*_(2, 94)_ = 163.65, *p* < 0.001, ηp^2^ = 0.78]. *Post-hoc* comparisons with Tukey HSD correction showed that musicians (*mean* = 9.66; *SD* = 0.75) had a significantly higher familiarity rating of the musical note than music learners (*mean* = 7.42; *SD* = 2.52) and non-musicians (*mean* = 2.24; *SD* = 1.84), while music learners had a significantly higher familiarity rating of the musical note than non-musicians.

To further explore the differential music expertise between musicians and music learners, the two groups completed self-report questions based on a 10-point Likert scale (i.e., 1 represents do not read music at all, and 10 represents being extremely confident in music reading and relevant knowledge). Musicians significantly outperformed music learners in sight-reading, with a large effect size [musicians: *mean* = 7.26, *SD* = 1.67, music learners: *mean* = 5.58, *SD* = 1.79; *t*_(60)_ = 3.75, *p* < 0.001, *d* = 0.97] and knowledge of music theory, with a medium effect size [musicians: *mean* = 6.16, *SD* = 1.67, music learners: *mean* = 5.08, *SD* = 1.74; *t*_(60)_ = 2.43, *p* = 0.018, *d* = 0.63]. In addition, musicians (*mean* = 5.58, *SD* = 7.72) spent more time in music reading per week than music learners, with a medium effect size [*mean* = 2.89, *SD* = 1.95; *t*_(44.15)_ = 2.07, *p* = 0.044, *d* = 0.49].

The participants of all three groups were matched in handedness and working memory. The Edinburgh Handedness Inventory (Oldfield, 1971; Cohen, 2008) was used to assess participants' handedness based on the 10-item (Oldfield, 1971) and 15-item versions (Cohen, 2008). No significant differences were observed among the three groups in the 10-item laterality index [musicians: *mean* = 74.87, *SD* = 35.46, music learners: *mean* = 66.73, *SD* = 46.78, non-musicians: *mean* = 66.06, *SD* = 44.93; *F*_(2, 94)_ = 0.48, *p* = 0.62] and 15-item augmented laterality index [musicians: *mean* = 73.60, *SD* = 34.20, music learners: *mean* = 65.51, *SD* = 44.79, non-musicians: *mean* = 64.95, *SD* = 42.98; *F*_(2, 94)_ = 0.50, *p* = 0.61]. Most participants were right-handed (musicians: *n* = 36, music learners: *n* = 22, non-musicians: *n* = 26), while a few of them were left-handed (musicians: *n* = 2, music learners: *n* = 2, non-musicians: *n* = 3) or with mixed-handedness (musicians: *n* = 0, music learners: *n* = 2, non-musicians: *n* = 4). Using participants with matched handedness was essential to rule out the possibility that the potential differences observed in hemispheric lateralization among musicians, music learners, and non-musicians were due to the actual difference in hemispheric lateralization *per se*, instead of an effect observed based on their music training background.

In addition, a set of *n*-back tasks comprised verbal and spatial two-back tasks was used to assess participants' verbal and spatial working memory. In the verbal two-back task, participants judged whether an English letter was the same as the one previously presented in a two-letter interval. While in the spatial two-back task, participants judged whether a symbol's location was the same as the location that was previously presented in a two-stimulus interval. Similarly, no significant differences were observed in the ACC [*F*_(2, 94)_ = 2.55, *p* = 0.08] and correct RT [*F*_(2, 93)_ =1.07, *p* = 0.35] in the set of *n*-back tasks, comprising a verbal and a spatial two-back task, among the three groups. Table 3 shows the participants' performance of the verbal and spatial two-back tasks respectively. Using participants with matched working memory could minimize the possibility that the potential differences observed in task performance among musicians, music learners, and non-musicians were due to their differences in working memory, instead of their music training background.

**Table 3:** The accuracy (ACC) and correct response time (RT) of the n-back tasks (verbal, spatial) among musicians, music learners, and non-musicians.

	**Accuracy (ACC)**			**Correct response time (RT)**		
	***M***	***SD***	***Range***	***M***	***SD***	***Range***
**a**. ***n*****-back task - verbal**						
Musicians	67.45	16.60	26.31–100.00	630.45	100.13	307.17–781.77
Music learners	56.78	15.46	28.95–89.47	617.05	84.14	434.17–782.06
Non-musicians	61.4	16.44	23.68–92.11	598.45	96.30	297.35–743.15
**b**. ***n*****-back task - spatial**						
Musicians	51.38	11.47	28.95–68.42	572.31	92.49	295.96–771.79
Music learners	48.58	13.60	10.53–65.79	598.24	84.29	370.72–731.11
Non-musicians	29.52	10.94	18.42–65.79	543.18	94.58	307.71–688.30

### 2.2 Materials

#### 2.2.1 Divided visual field sequential matching tasks

Four sets of musical elements, namely pitch elements (i.e., pitch, harmony), temporal elements (i.e., rhythm), and expressive elements (i.e., articulation), were included in the divided visual field sequential matching tasks. All musical elements were created using Sibelius (Avid Technology Inc., USA) and PhotoScapeX (MOOII Tech, Korea) in 2,400 dpi.

**Figure 1:**
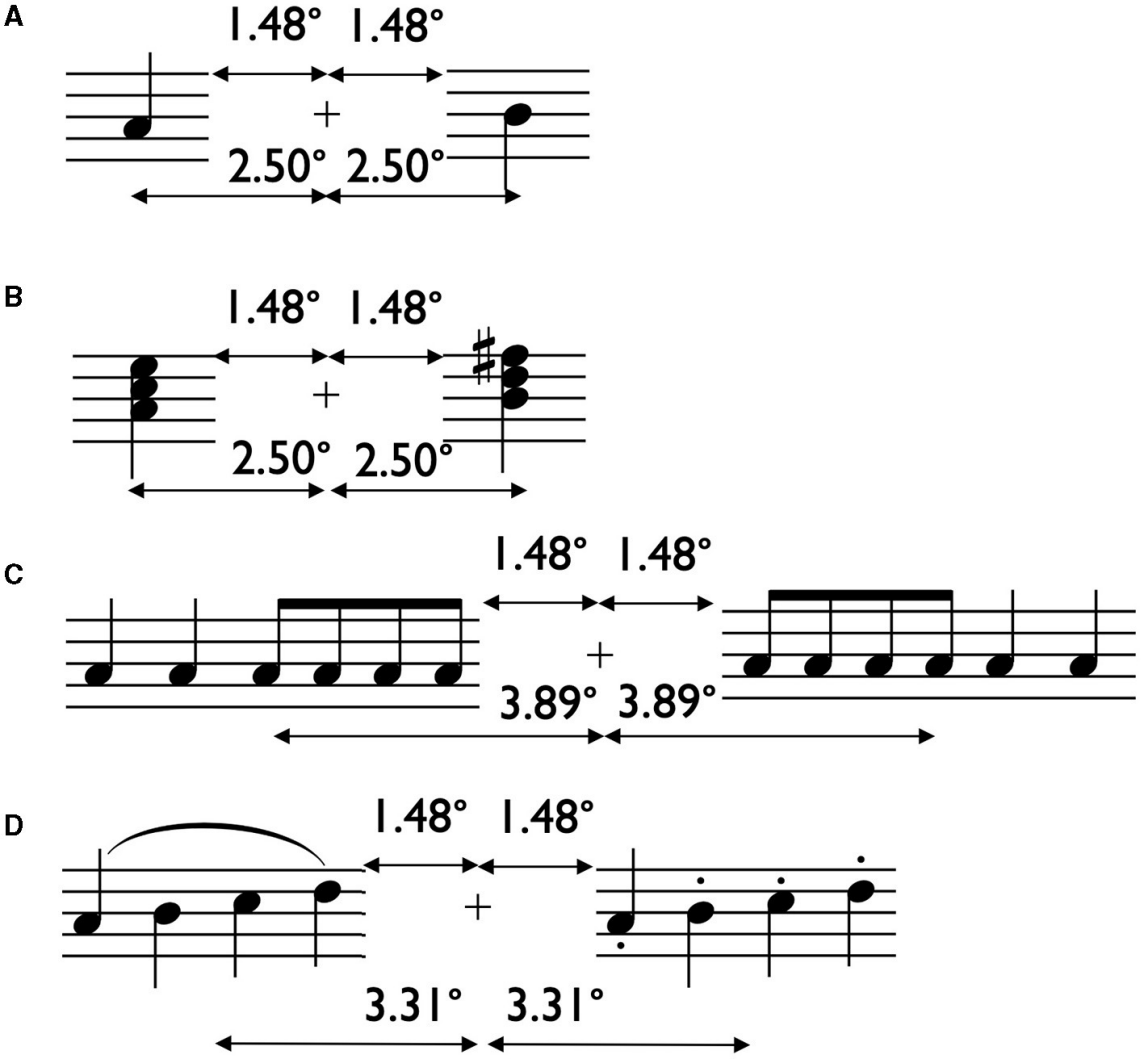
The presentation position of pitch elements [**(A)** pitch, **(B)** harmony], temporal elements [**(C)** rhythm], and expressive elements [**(D)** articulation] in the divided visual field sequential matching task. The vertical edge of all stimuli was a visual angle of 1.48° toward the left/right from the center of the screen. **(A, B)** For pitch and chord, the stimulus center was a visual angle of 2.5° toward the left/right from the center of the screen. **(C)** For rhythm, the stimulus center was a visual angle of 3.89° toward the left/right from the center of the screen. **(D)** For articulation, the stimulus center was a visual angle of 3.31° toward the left/right from the center of the screen.

The size of musical elements (e.g., a crotchet) included in the divided visual field sequential matching tasks was approximately a double of a crotchet (i.e., 12 mm × 10 mm) from the *Specimen Aural Tests* for Grades 1–5 for Practical exams, published by the Associated Board of The Royal Schools of Music (ABRSM) in print, at a normal viewing distance of 30 cm. This ensures the readability of musical elements presented at a viewing distance of 60 cm in tasks. With its corresponding five-line staff, each single note and chord stimulus subtended a horizontal and vertical visual angle of 1.40° × 2.69°. Each rhythmic stimulus subtended a horizontal and vertical visual angle of 4.18° × 2.69°, while each articulatory stimulus subtended a horizontal and vertical visual angle of 3.03° × 2.69°. The vertical edge of all stimuli was a visual angle of 1.48° toward the left or the right away from the center (see Figure 1 for more information).

##### 2.2.1.1 Pitch

**Figure 2:**
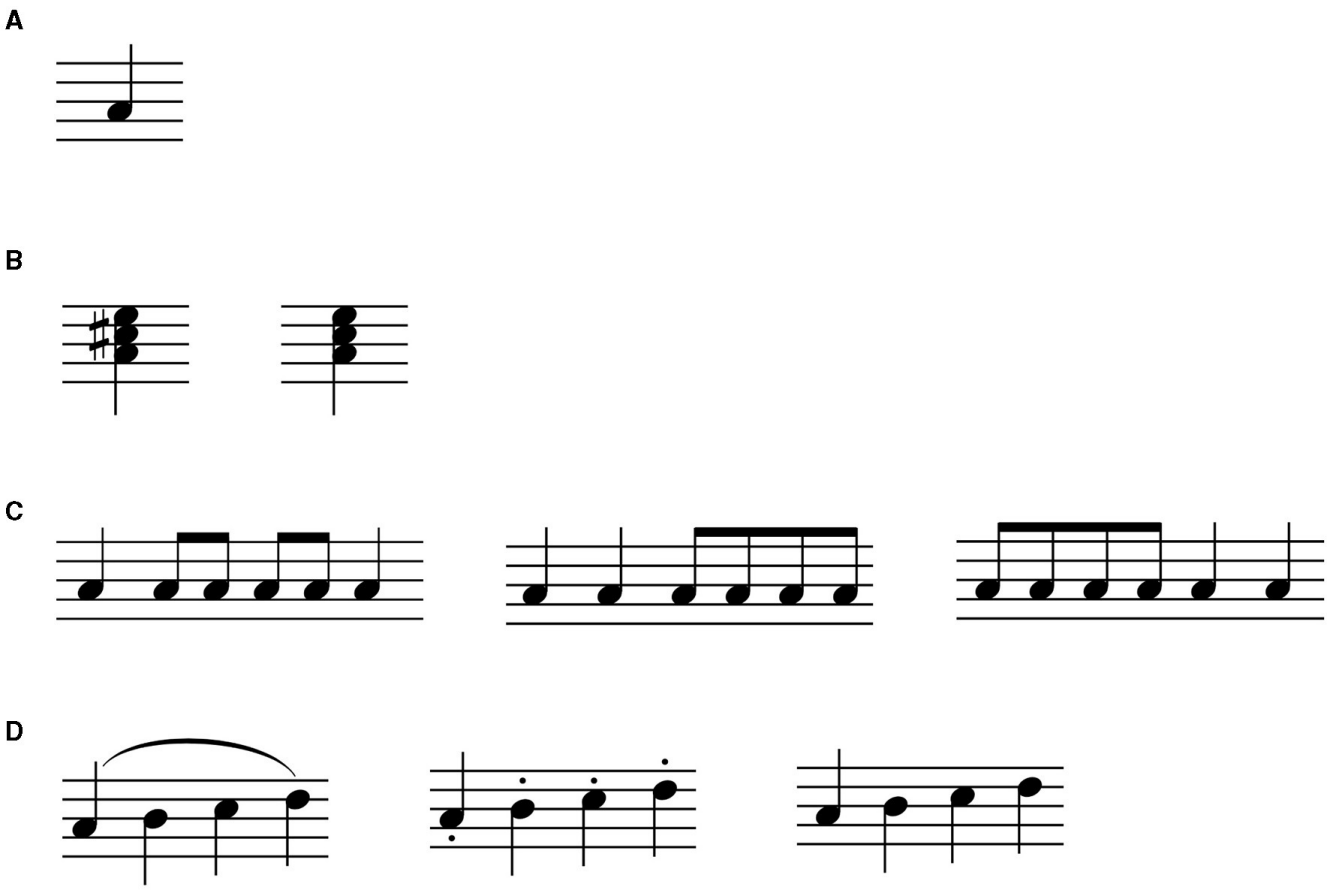
Sample stimuli of pitch elements [**(A)** pitch, **(B)** harmony], temporal elements [**(C)** rhythm], and expressive elements [**(D)** articulation] used in the divided visual field sequential matching task. **(A)** Refers to a single note A_4_ in crotchet (1 beat). **(B)** Refers to two chords (i.e., triads) with the root of A_4_ in crotchet, including an A major chord (left) and an A minor chord (right). **(C)** Refers to three rhythmic patterns in note A_4_, including pattern 1 (left): 1 crotchet, 4 semiquavers (half beats), 1 crotchet; pattern 2 (middle): 2 crotchets, 4 semiquavers; pattern 3 (right): 4 semiquavers, 2 crotchets. **(D)** Refers to three articulatory patterns with ascending pitches starting from A_4_, including pattern 1 (left): with slur; pattern 2 (middle): with staccato; pattern 3 (right): no articulation.

**Figure 3:**
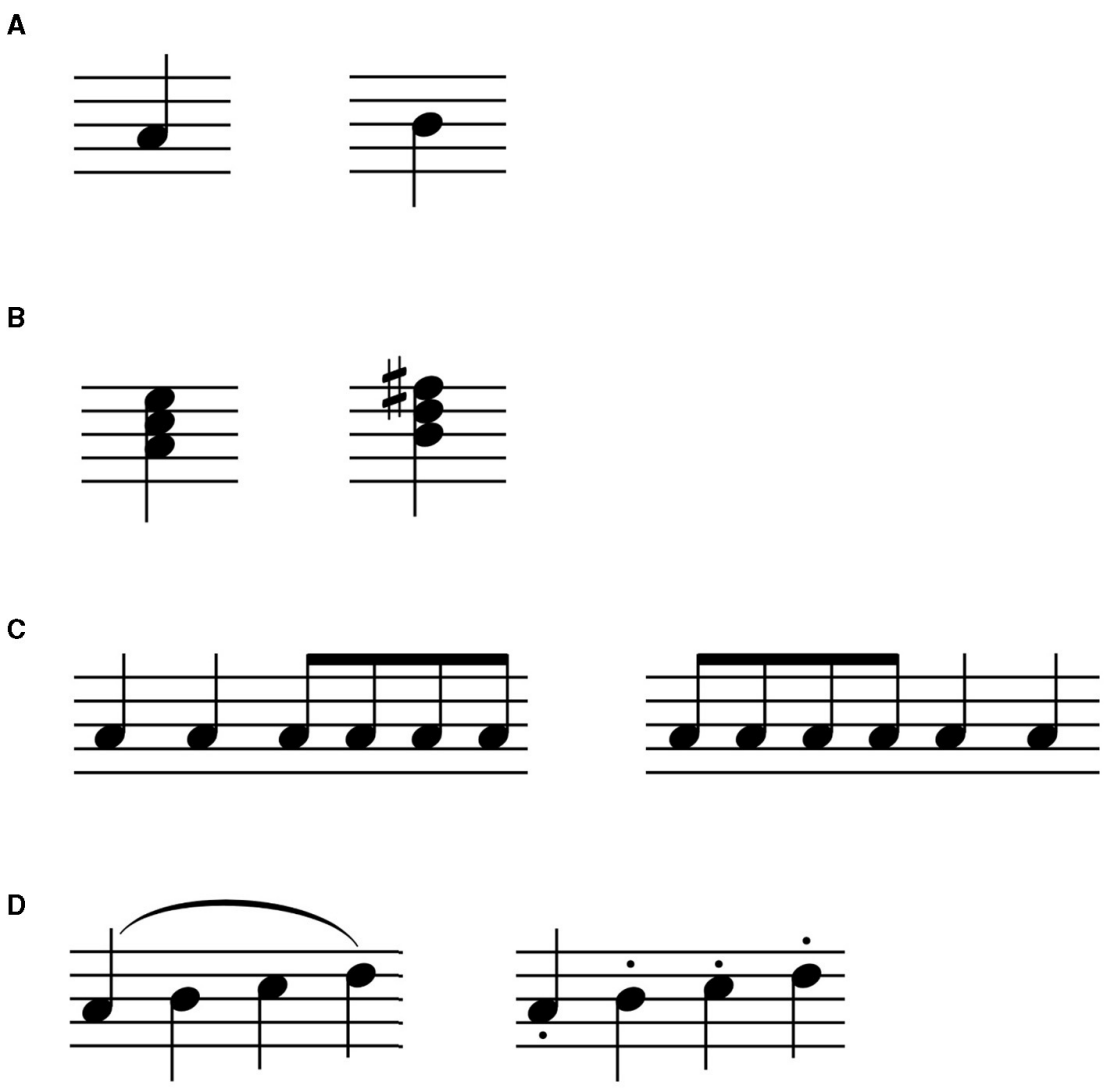
Sample stimulus pairs of pitch elements [**(A)** pitch, **(B)** harmony], temporal elements [**(C)** rhythm], and expressive elements [**(D)** articulation] used in the different trials of the divided visual field sequential matching task. **(A)** Refers to a stimulus pair of pitch (e.g., left - A_4_ vs. right - B_4_), which has an upper major 2^nd^ difference between the two target stimuli. **(B)** Refers to a stimulus pair of harmony (e.g., left - A minor chord in root position vs. right - B minor chord in root position), which shows an upper major 2^nd^ difference between the two target stimuli. **(C)** Refers to a stimulus pair of rhythm (e.g., left - 2 crotchets, 4 semiquavers vs. right - 4 semiquavers, 2 crotchets), which has different rhythmic patterns between the two target stimuli. **(D)** Refers to a stimulus pair of articulation (e.g., left - with slur vs. right - with staccato), which shows different articulatory patterns between the two target stimuli.

Single notes ranging from G3 to E6 (*n* = 20) were created with crotchets (1 beat; see Figure 2A). Experimental trials included single notes from A3 to D6, while the remaining stimuli were for practice trials only. This was to avoid any possible exposure of particular notes before the experimental session. Same trials referred to trials with identical target stimuli, while different trials referred to trials either with an upper or lower major 2^nd^ or major 3^rd^ difference between the first and second target stimuli (see Figure 3A). The minimal visual differences observed from the first and second target stimuli among different trials enabled a sensitive measurement of music reading.

##### 2.2.1.2 Harmony

Chords (i.e., triads) with the roots of G3 to B5 (*n* = 34) were created with crotchets (1 beat; see Figure 2B). Both major (*n* = 17) and minor (*n* = 17) triads were included. Experimental trials included triads rooted from D4 to G5, while the remaining stimuli were for practice trials only. Only major or minor triads were used in each trial. Same trials referred to trials with identical target stimuli, while different trials referred to trials with two triads that differ in the roots with either an upper or lower major 2^nd^ or major 3^rd^ difference between the first and second target stimuli (see Figure 3B). The minimal visual differences observed from the first and second target stimuli among different trials enabled a sensitive measurement of music reading.

##### 2.2.1.3 Rhythm

Rhythmic patterns in 4/4 time signature, ranging from G3 to E6 (*n* = 60), were created. Three sets of rhythmic patterns with common rhythmic groupings included pattern 1 (*n* = 20; see Figure 2C left): 1 crotchet, 4 semiquavers (half beat), 1 crotchet; pattern 2 (*n* = 20; see Figure 2C middle): 2 crotchets, 4 semiquavers; pattern 3 (*n* = 20; see Figure 2C right): 4 semiquavers, 2 crotchets. The same pitch was used for all notes in each stimulus. Experimental trials included rhythmic patterns ranging from A3 to D6, while the remaining stimuli were for practice trials only. Same trials referred to trials with identical target stimuli, while different trials referred to trials with different rhythmic patterns between the first and second target stimuli (see Figure 3C). No time signature was shown in the stimuli to align with the presentation of musical elements in other blocks.

##### 2.2.1.4 Articulation

Articulatory patterns in 4/4 time signature, ranging from G3 to E6 (*n* = 60) were created. Three sets of articulatory patterns included pattern 1 (*n* = 20): with slur (see Figure 2D left), pattern 2 (*n* = 20): with staccato (see Figure 2D middle), and pattern 3 (*n* = 20): no articulation (see Figure 2D right). The same set of ascending pitches was used across the three articulatory patterns. Experimental trials included articulatory patterns ranging from A3 to D6, while the remaining stimuli were for practice trials only. Same trials referred to trials with identical target stimuli, while different trials referred to trials with different articulatory patterns between the first and second target stimuli (see Figure 3D). No time signature was shown in the stimuli to align with the presentation of musical elements in other tasks.

#### 2.2.2 Music expertise tasks

The music expertise tasks comprised a visual memory (16 trials) and a visual-auditory music tasks (16 trials) to assess participants' music expertise. In total, 4-bar musical phrases were selected from the soprano and alto voices in four-part chorales (i.e., SATB vocal repertories) composed by J. S. Bach (*n* = 32). Diatonic phrases (*n* = 16) in common keys, either in G major, D major, F major, or Bb major, with common cadences, either ended in I or V chords, were selected as the experimental trials. Non-diatonic phrases (*n* = 16) were created based on the alternative set of diatonic phrases from the same source with bars shuffled, and one accidental altered in each bar. This was to ensure that no exact musical structures were shared between the diatonic and non-diatonic phrases, which might influence participants' performance in task. The size of musical elements (e.g., a crotchet) included in music expertise tasks was the same as those in the divided visual field sequential matching tasks.

In the visual memory task, a set of diatonic phrases (*n* = 8) and non-diatonic phrases (*n* = 8) were included. A set of single bars (*n* = 16) was created to serve as probes. Same trials referred to trials that the probe was identical to one of the bars that was previously shown in the musical phrase. Different trials referred to trials that the probe contains one different note as compared to one of the bars that was previously shown in the musical phrase.

In the visual-auditory music task, a set of visually shown diatonic phrases (*n* = 8) and non-diatonic phrases (*n* = 8) were included. A set of auditory soundtracks corresponding to the 4-bar musical phrases (*n* = 16) was created using the sound of piano to serve as probes using Sibelius (Avid Technology Inc., USA). Same trials referred to trials that the auditory probe was identical to the visually shown musical phrase. Different trials referred to trials that the auditory probe contained one different note as compared to the visually shown musical phrase.

#### 2.2.3 *n*-back tasks

The set of *n*-back tasks comprised verbal (40 trials) and spatial two-back tasks (40 trials) to assess participants' verbal and spatial working memory. In the verbal two-back task, English letters (*n* = 10) were used as stimuli. In the spatial two-back task, symbols (*n* = 10), including some Russian letters which do not carry any meanings to Cantonese-speaking participants, were used as stimuli.

#### 2.2.4 Questionnaires

A demographic questionnaire was used to assess participants' demographics, including language, educational background, visual impairments, auditory impairments, and music training background. In addition, Goldsmiths Musical Sophistication Index (Gold-MSI; Müllensiefen et al., 2014) with 38 items was used to measure participants' musicality. Also, the Edinburgh Handedness Inventory (Oldfield, 1971; Cohen, 2008) was used to assess participants' handedness based on the 10-item (Oldfield, 1971) and 15-item versions (Cohen, 2008).

### 2.3 Design

The experimental design of this study comprise a set of divided visual field sequential matching tasks with a 4 × 2 × 3 mixed design. It included two within-subject variables [musical elements: pitch, harmony, rhythm, and articulation; visual fields (VFs): LVF vs. RVF] and one between-subject variable (groups: musicians vs. music learners vs. non-musicians).

The divided visual field sequential matching task examines hemispheric lateralization as a behavioral technique, with its theoretical basis based on Bourne (2006), in which suggested the contralateral relationships between the visual fields and hemispheres. For example, the stimulus shown in the right visual field projects more directly to the left hemisphere (RVF/LH), while the stimulus shown in the left visual field projects more directly to the right hemisphere (LVF/RH).

Additionally, the divided visual field sequential matching task paradigm was designed based on the recommendations in Hunter and Brysbaert (2008) for naming tasks. For instance, the current study consists of sufficient trials, as shown in the blocks with pitch (224 trials), harmony (224 trials), rhythm (216 trials), and articulation (208 trials). Each stimulus was shown with an equal number of occurrences in the LVF and RVF. No stimulus degradation was applied. Participants' central fixation was controlled and monitored using an eye-tracker. A bilateral presentation of stimuli for 200 ms was used to assess hemispheric lateralization. The design was based on the recommendation stated in Hunter and Brysbaert (2008) to limit voluntary saccade that has 340 ms average onset latency (Walker and McSorley, 2006, as cited in Hunter and Brysbaert, 2008). Presenting the stimuli for 200 ms could ensure participants' central fixation during bilateral stimuli presentation, and thus result in an accurate measurement of hemispheric lateralization using the divided visual field sequential matching task. Last but not least, masks were also applied after stimulus offset.

The computerized divided visual field sequential matching tasks were conducted using Eprime 3.0 (Psychology Software Tools, Pittsburgh, PA, USA) for stimulus presentation with a 24″ screen with a resolution of 1,920 × 1,080. A Tobii Pro Spectrum eye-tracker (Tobii, Danderyd, Sweden) is a high-frequency screen-based eye-tracker that used to monitor participants' central fixation with binocular eye-tracking with a sampling frequency at 600 Hz. A green rectangle was shown if the participant has fixated at the central fixation for 250 ms as detected by the eye-tracker, prior to the 200 ms bilateral presentation of musical elements in the LVF and RVF. The 250 ms fixation detection threshold was set with respect to the average fixation duration of normal reading (Rayner, 1993). A chin and forehead rest (i.e., SR Research Head Support; SR-Research Ltd., Mississauga, Ontario, Canada) was used to minimize participants' head movement during the tasks at a viewing distance of 60 cm. A nine-point binocular calibration was performed before the start of each block. Recalibration was conducted if the initial calibration was unsatisfactory or unsuccessful. All blocks were counterbalanced with randomized trials. An equal number of same and different trials was arranged. For different trials, the only difference between the first and second target stimuli was either in pitch, harmony, rhythm, and or articulation in each separate block respectively, while other musical elements were all controlled.

Participants indicated their responses on a Chronos response box (Psychology Software Tools, Pittsburgh, PA, USA) for all tasks using both index and middle fingers. Fingerings were counterbalanced among participants. This design was to avoid the possible lateralization effects induced by single-hand responses (Mohr et al., 1994).

This study sought the Ethical Review for Research involving Human Participants as Research Subjects from the Research Ethics Committee (REC), Hong Kong Metropolitan University, and was conducted in accordance to the ethical guidelines stated in the World Medical Association's Declaration of Helsinki.

### 2.4 Procedures

Each experimental session was conducted in the Experimental room of the Psychology and Behavioral Sciences Laboratory at the Hong Kong Metropolitan University, Hong Kong, China. Participants were recruited through mass email and poster promotions within the University and referrals. A few preliminary screening questions regarding music training background were applied when participants signed up for the study.

On the day of experiment, the experimenter checked once again with participants regarding their music training backgrounds upon arrival. Participants who fulfilled the inclusion criteria of musicians, music learners, and non-musicians would then proceed to the study. A short briefing regarding the experimental procedures was given to each participant. A written consent was obtained before the start of each experimental session. Each experimental session lasted for about 1.5 h.

Each experimental session comprised a set of computerized divided visual field sequential matching tasks. Each block, namely pitch, harmony, rhythm, and articulation, consisted of four sections respectively. In addition, three questionnaires [i.e., a demographic questionnaire, Goldsmiths Musical Sophistication Index (Gold-MSI; Müllensiefen et al., 2014), and Edinburgh Handedness Inventory (Oldfield, 1971; Cohen, 2008)] and two computerized preliminary tasks (i.e., *n*-back tasks and music expertise tasks) were included.

**Figure 4:**
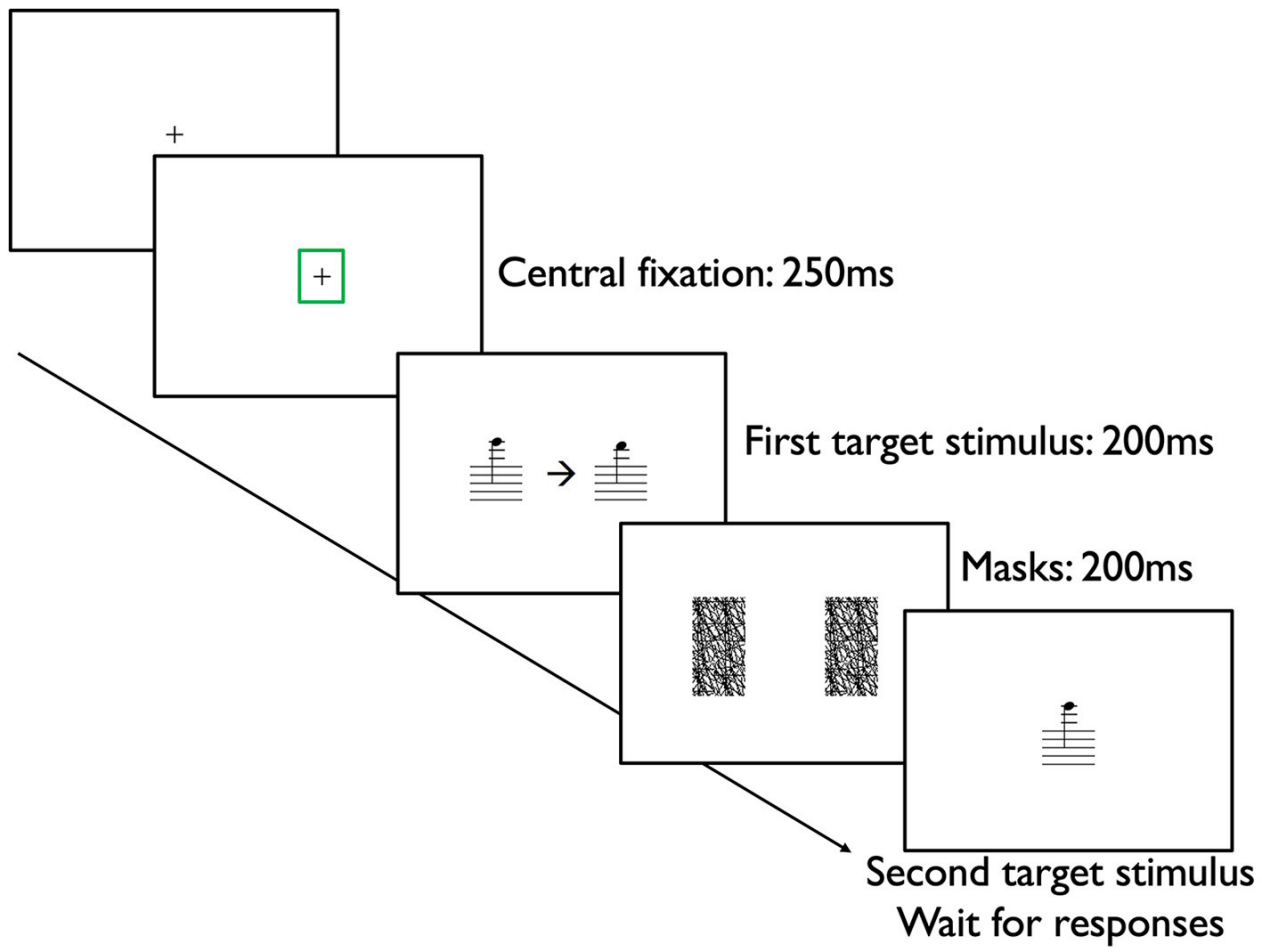
Procedures of the divided visual field sequential matching task. This figure demonstrates a sample trial from the pitch block, in which participants first fixated at the central fixation (+) for 250 ms, indicating by a green rectangle surrounding the central fixation as measured by an eye-tracker. Then, two single notes in crotchets (1 beat) were presented simultaneously at the left visual field (LVF) and right visual field (RVF) for 200 ms, together with a central arrow either pointing to the left (←) or right ( → ), specifying the first target stimulus. Two masks were then presented for 200 ms in the LVF and RVF to avoid formation of afterimages. Participants then judged whether the second target stimulus presenting at the center of the screen is the same or not as the first target stimulus by pressing a response box.

The divided visual field sequential matching tasks were conducted on Eprime 3.0 (Psychology Software Tools, Pittsburgh, PA, USA). For each trial, participants first fixated at the center of the screen (+). A Tobii Pro Spectrum eye-tracker (Tobii, Danderyd, Sweden) was used to monitor participants' central fixation. For each trial, participants were instructed to fixate at the center of the screen. When their eye gazes stayed at the central fixation for 250 ms, a green rectangle surrounding the central fixation emerged. Then, two stimuli (pitch/harmony/rhythm/articulation in each respective block) were presented briefly and simultaneously for 200 ms on the left visual field (LVF) and right visual field (RVF). A central arrow either pointing to the left (←) or right ( → ) was also simultaneously presented for 200 ms, indicating the first target stimulus. Masks were then presented for 200 ms to avoid afterimages being formed. Then, one stimulus (pitch/harmony/rhythm/articulation in each respective block) was presented at the center of the screen as the second target stimulus and waited for participants' responses (Figure 4). Participants judged whether the first target stimulus was the same as the second target stimulus by pressing the Chronos response box (Psychology Software Tools; Pittsburgh, PA, USA) as quickly and accurately as possible. ACC and RT were recorded.

Besides, the demographic questionnaire and Goldsmiths Musical Sophistication Index (Gold-MSI; Müllensiefen et al., 2014) were conducted on Qualtrics (Qualtrics, Provo, UT, USA). The Edinburgh Handedness Inventory (Oldfield, 1971; Cohen, 2008) was conducted on Cohen (2008) webpage, https://www.brainmapping.org/shared/Edinburgh.php#. The sets of *n*-back tasks and music expertise tasks were carried out on Eprime 3.0 (Psychology Software Tools, Pittsburgh, PA, USA). A few practice trials were provided for all computerized tasks before its respective experimental tasks. Short breaks were provided between sections within each block.

A debriefing was delivered to the participants upon the completion of all tasks. A small honorarium was given to the participants to thank them for their support.

## 3 Results

The participants' ACC and correct RT in the divided visual field sequential matching tasks were analyzed using IBM SPSS Statistics for Windows, Version 26.0 (Armonk, NY). Correct RT refers to the RT of correct trials. In addition, the RTs with values ± 3SD were considered as outliers, and were thus be removed from the analyses. Prior to the statistical analyses, the Kolmogorov-Smirnov test was used to examine the normality of participants' ACC and correct RT data in the divided visual field sequential matching tasks across the three groups. Only musicians' ACC was significantly different from a normal distribution [*D* (38) = 0.18, *p* = 0.003], while music learners' ACC [*D* (26) = 0.14, *p* = 0.20] and non-musicians' ACC [*D* (33) = 0.11, *p* = 0.20] were normally distributed. Musicians' correct RT [*D* (38) = 0.11, *p* = 0.20], music learners' correct RT [*D* (26) = 0.16, *p* = 0.08], and non-musicians' correct RT [*D* (33) = 0.14, *p* = 0.10] were normally distributed. Thus, based on the results from the Kolmogorov-Smirnov tests, parametric tests were used in the subsequent analyses.

A Repeated Measures Analysis of Variance (ANOVA) was conducted based on a 4 × 2 × 3 mixed design, including two within-subject variables [musical elements: pitch, harmony, rhythm, and articulation; visual fields (VFs): LVF vs. RVF] and one between-subject variable (Groups: musicians vs. music learners vs. non-musicians). The visual field with a comparatively higher accuracy and/or faster response time than the other visual field suggests a corresponding visual field advantage. The visual field advantage could further be interpreted based on the contralateral relationships between the visual fields (VFs) and hemispheres, as represented by the right visual field-left hemisphere (RVF/LH) and left visual field-right hemisphere (LVF/RH) (Bourne, 2006), for its corresponding hemispheric lateralization effect. Table 4 shows the descriptive statistics of the ACC and correct RT of the divided visual field matching tasks among musicians, music learners, and non-musicians.

**Table 4:** The accuracy (ACC) and correct response time (RT) of the divided visual field matching task among musicians, music learners, and non-musicians.

	**Accuracy (ACC)**			**Correct response time (RT)**		
	***M***	***SD***	***Range***	***M***	***SD***	***Range***
**a. Divided visual field matching task – pitch (LVF)**						
Musicians	80.94	11.14	55.35–97.32	782.81	223.35	440.84–1,261.27
Music learners	70.78	15.04	33.04–92.86	833.97	292.45	494.60–1,511.08
Non-musicians	62.31	10.24	44.64–87.50	714.67	237.32	373.51–1,576.88
**b. Divided visual field matching task – pitch (RVF)**						
Musicians	81.91	16.99	16.07–98.21	778.27	230.97	508.06–1,333.06
Music learners	72.97	13.57	41.96–95.54	817.27	258.80	451.98–1,357.56
Non-musicians	61.71	9.50	44.64–82.14	757.13	262.48	344.14–1,604.34
**c. Divided visual field matching task – harmony (LVF)**						
Musicians	84.14	12.07	51.78–97.32	736.14	169.04	390.79–1,181.61
Music learners	78.61	14.23	38.39–96.43	837.69	276.74	530.93–1,649.07
Non-musicians	75.24	13.44	48.21–91.07	680.45	184.47	379.09–1,351.42
**d. Divided visual field matching task – harmony (RVF)**						
Musicians	80.90	19.45	8.04–99.11	765.06	209.95	341.04–1,275.58
Music learners	78.16	17.25	15.18–96.43	812.70	251.94	482.40–1,634.26
Non-musicians	71.29	13.60	44.64–91.07	717.55	223.82	295.84–1,521.44
**e. Divided visual field matching task – rhythm (LVF)**						
Musicians	74.03	15.97	26.85–97.22	844.88	215.17	383.28–1,289.92
Music learners	68.55	16.10	36.11–95.37	857.84	352.21	491.20–1,990.65
Non-musicians	66.11	14.80	46.29–96.30	706.80	287.31	276.26–1,699.21
**f. Divided visual field matching task – rhythm (RVF)**						
Musicians	70.61	17.81	13.89–93.52	841.30	237.30	326.17–1,316.36
Music learners	68.52	15.92	37.04–89.81	921.46	341.19	486.98–1,938.30
Non-musicians	64.17	13.68	40.74–91.67	726.64	314.04	269.92–1,681.75
**g. Divided visual field matching task – articulation (LVF)**						
Musicians	86.63	11.62	49.07–100.00	772.87	205.36	460.07–1,420.68
Music learners	82.43	16.40	19.63–99.08	813.93	249.59	481.70–1,316.66
Non-musicians	76.02	16.41	41.12–98.17	685.64	250.28	107.04–1,631.32
**h. Divided visual field matching task – articulation (RVF)**						
Musicians	88.24	18.10	10.28–100.00	756.96	239.59	517.63–1,615.40
Music learners	84.56	23.06	9.34–100.00	747.73	208.22	378.40–1,182.63
Non-musicians	78.06	17.67	30.84–100.00	706.08	265.10	157.60–1,639.17

### 3.1 Repeated measures analysis of variance (ANOVA) on the ACC data

**Figure 5:**
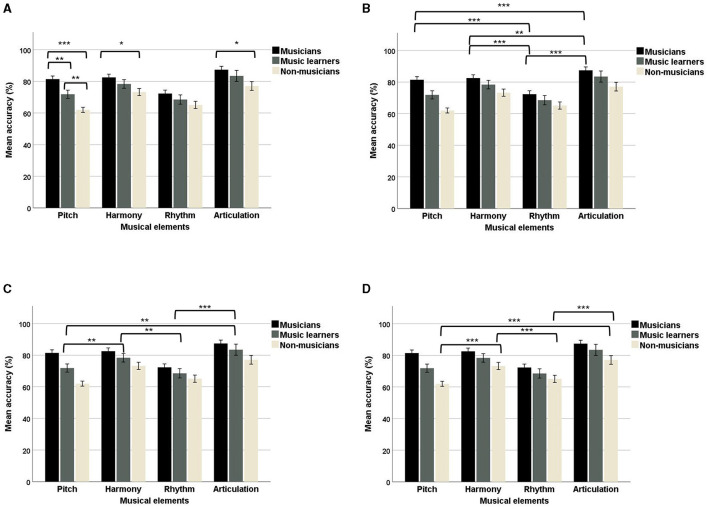
Mean accuracy of the four musical elements (pitch, harmony, rhythm, and articulation) examined in the divided visual field sequential matching tasks among musicians, music learners, and non-musicians (error bars show one standard error; *p < 0.05, **p < 0.01, ***p < 0.001). **(A)** Represents the results of among the four musical elements. **(B**–**D)** Represent the results related to musicians, music learners, and non-musicians respectively.

In the ACC data, a significant two-way interaction between musical elements and groups was observed, *F*_(6, 186)_ = 4.57, *p* < 0.001, ηp^2^ = 0.13, suggesting that musicians, music learners, and non-musicians performed differently in the four musical elements, with a medium effect size. To further understand this two-way interaction, we examined the data on different musical elements (Figure 5A). In terms of pitch, musicians (*mean* = 81.42; *SD* = 12.14) significantly outperformed music learners (*mean* = 71.88; *SD* = 13.22) and non-musicians (*mean* = 62.01; *SD* = 9.16). Music learners also significantly outperformed non-musicians. Regarding harmony, musicians (*mean* = 82.52; *SD* = 12.84) significantly outperformed non-musicians (*mean* = 73.27; *SD* = 13.09), but not music learners (*mean* = 78.38; *SD* = 13.81). For rhythm, no significant differences were observed among musicians, music learners, and non-musicians. In terms of articulation, musicians (*mean* = 87.40; *SD* = 13.18) significantly outperformed non-musicians (*mean* = 77.06; *SD* = 15.84), but not music learners (*mean* = 83.48; *SD* = 17.92). In addition, we also examined the data based on musicians, music learners, and non-musicians. For musicians, a significant main effect of musical elements was observed, *F*_(3, 35)_ = 43.38, *p* < 0.001, ηp^2^ = 0.79, suggesting that pitch (*mean* = 81.43; *SD* = 12.13) and harmony (*mean* = 82.52; *SD* = 12.84) had similar ACC, as supported by a large effect size. However, rhythm (*mean* = 72.32; *SD* = 13.43) had a lower ACC than pitch, harmony, and articulation (*mean* = 87.38; *SD* = 13.19; Figure 5B). In contrast, articulation had a higher ACC than pitch, harmony and articulation. For music learners, a significant main effect of musical elements, with a large effect size, was observed, *F*_(3, 23)_ = 9.69, *p* < 0.001, ηp^2^ = 0.56, suggesting that pitch (*mean* = 71.88; *SD* = 13.22) had a lower ACC than harmony (*mean* = 78.38; *SD* = 13.81), and articulation (*mean* = 83.49; *SD* = 17.92), but not rhythm (*mean* = 68.53; *SD* = 14.90). Harmony had a higher ACC than rhythm, while rhythm had a lower ACC than articulation (Figure 5C). For non-musicians, a significant main effect of musical elements with a large effect size was observed, *F*_(3, 30)_ = 25.52, *p* < 0.001, ηp^2^ = 0.72, suggesting that pitch (*mean* = 62.01; *SD* = 9.16) had a lower ACC than harmony (*mean* = 73.27; *SD* = 13.09) and articulation (*mean* = 77.06; *SD* = 15.84), but not rhythm (*mean* = 65.13; *SD* = 13.09). Harmony had a higher ACC than rhythm, while rhythm had a lower ACC than articulation (Figure 5D).

**Figure 6:**
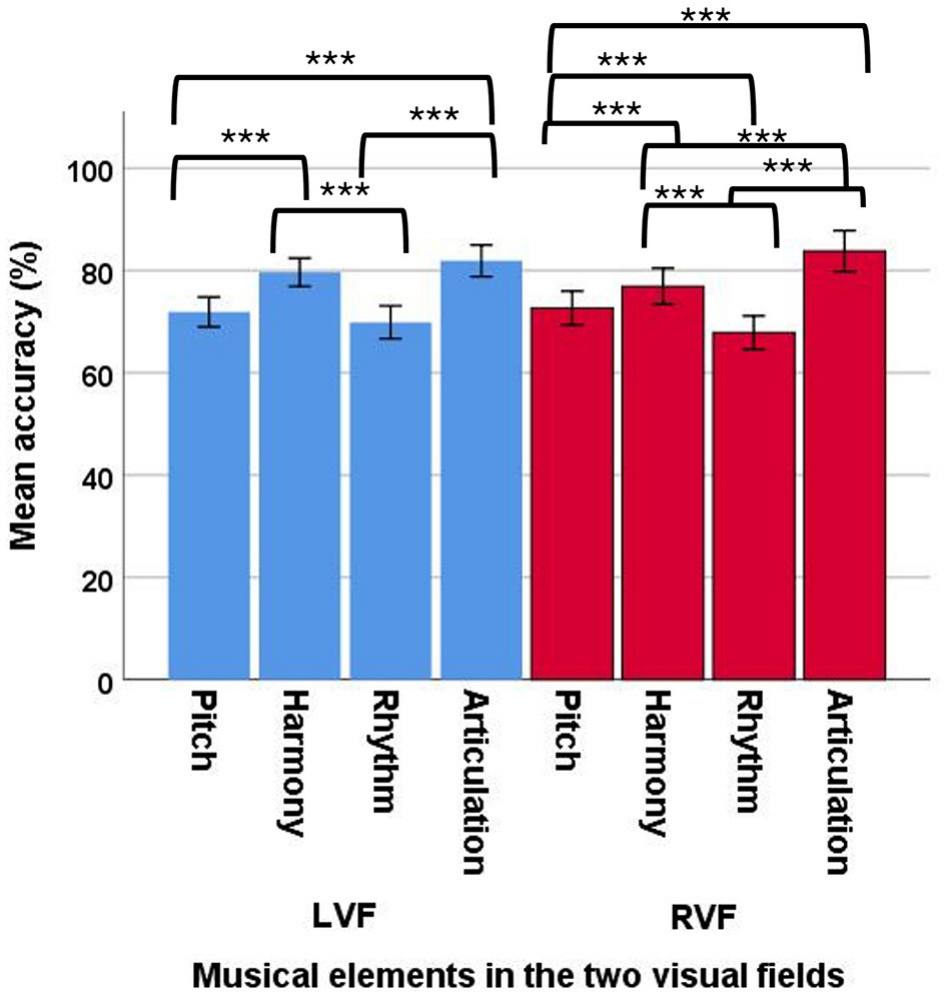
Mean accuracy of the four musical elements (pitch, harmony, rhythm, and articulation) examined in the divided visual field sequential matching tasks across the two VFs (LVF and RVF; error bars show one standard error; ***p < 0.001).

In addition, a significant two-way interaction between musical elements and VFs was observed, *F*_(3, 92)_ = 4.54, *p* = 0.005, ηp^2^ = 0.13, suggesting that different musical elements had different VF processing advantages, with a medium effect size. To further understand this two-way interaction, we examined the data on different musical elements (Figure 6). No significant main effects of VF were observed for pitch [*F*_(1, 94)_ = 0.48, *p* = 0.49], harmony [*F*_(1, 94)_ = 2.65, *p* = 0.11], rhythm [*F*_(1, 94)_ = 1.24, *p* = 0.27], and articulation [*F*_(1, 94)_ = 1.57, *p* = 0.21], suggesting no particular processing advantages were found in either the LVF or RVF for each musical element. In addition, we examined the data from separate VFs for the four musical elements. A significant main effect of musical elements was observed in the LVF, *F*_(3, 92)_ = 39.09, *p* < 0.001, ηp^2^ = 0.56, suggesting that the four musical elements showed different ACC values in the LVF, with a large effect size. The *post-hoc* comparisons showed that pitch (*mean* = 71.34; *SE* = 1.24) had a significantly lower ACC than harmony (*mean* = 79.33; *SE* = 1.35) and articulation (*mean* = 81.68; *SE* = 1.51), but not rhythm (*mean* = 69.33; *SE* = 1.61). Harmony had a higher ACC than rhythm, and rhythm had a lower ACC than articulation. In addition, a significant main effect of musical elements was also observed in the RVF, *F*_(3, 92)_ = 41.69, *p* < 0.001, ηp^2^ = 0.58, suggesting that the four musical elements showed different ACC values in the RVF, with a large effect size. The *post-hoc* comparisons showed that rhythm (*mean* = 67.77; *SE* = 1.64) had a significantly lower ACC than pitch (*mean* = 72.20; *SE* = 1.43), harmony (*mean* = 76.78; *SE* = 1.75), and articulation (*mean* = 83.61; *SE* = 2.00). In addition, articulatory processing had a significantly higher ACC than pitch, harmony, and rhythm. Harmony also had a higher ACC than pitch.

**Figure 7:**
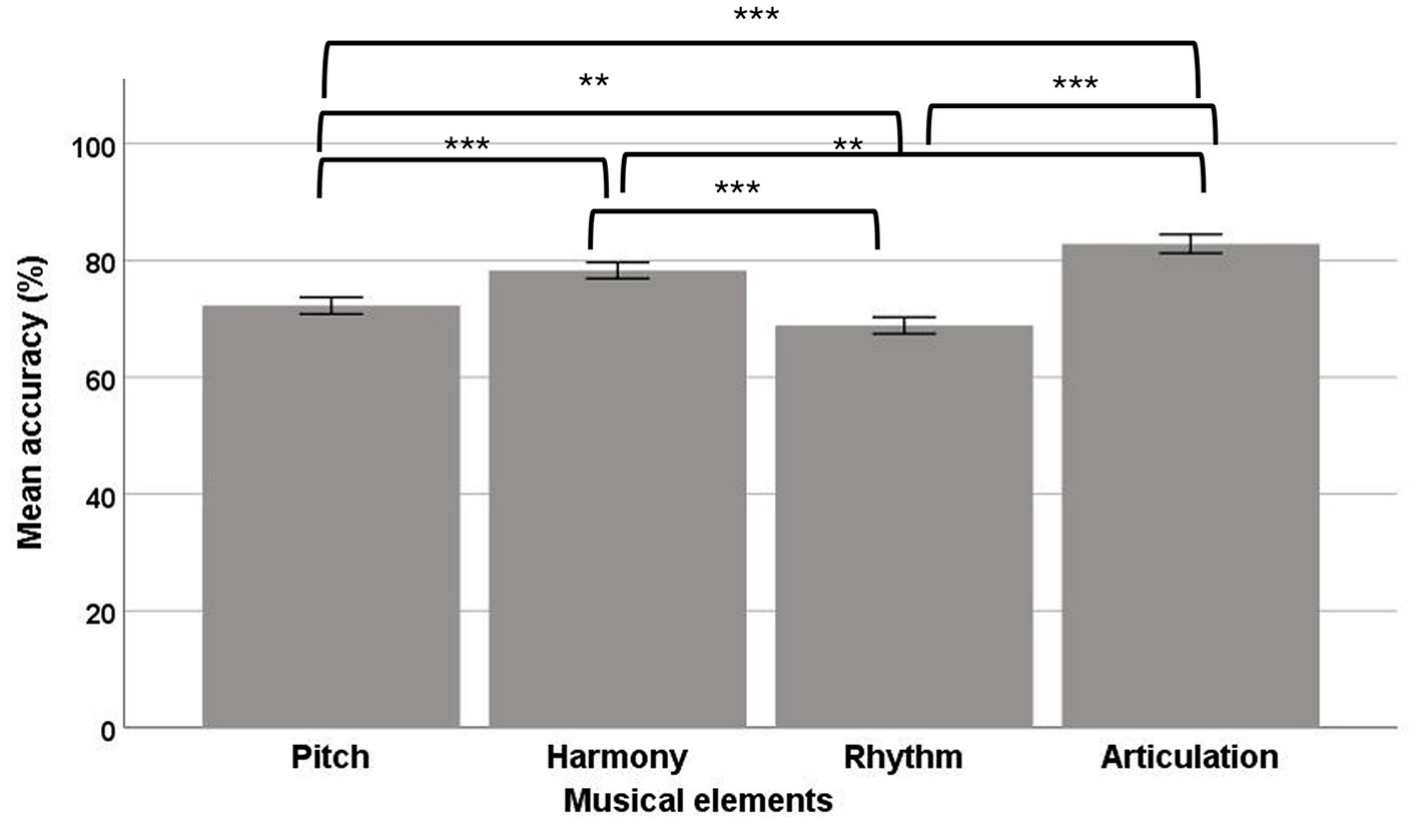
Mean accuracy of the four musical elements (pitch, harmony, rhythm, and articulation) examined in the divided visual field sequential matching tasks (error bars show one standard error; **p < 0.01, ***p < 0.001).

In the ACC data, a significant main effect of musical elements was observed [*F*_(3, 92)_ = 60.11, *p* < 0.001, ηp^2^ = 0.66], suggesting the four musical elements had different ACC values, with a large effect size. The *post-hoc* comparisons showed that rhythm (*mean* = 68.66; *SE* = 1.41) had a significantly lower ACC than pitch (*mean* = 71.77; *SE* = 1.19), harmony (*mean* = 78.06; *SE* = 1.36), and articulation (*mean* = 82.65; *SE* = 1.59; Figure 7). In addition, articulation has also showed a significantly higher ACC than pitch, harmony, and rhythm. Harmony also had a higher ACC than pitch. No other significant comparisons were observed.

A significant main effect of groups was also observed [*F*_(2, 94)_ = 8.69, *p* < 0.001, ηp^2^ = 0.16], suggesting that musicians, music learners, and non-musicians had different ACC values, with a large effect size. The post-hoc comparisons showed that musicians (*mean* = 80.91; *SD* = 11.77) and music learners (*mean* = 75.57; *SD* = 12.19) had a significantly higher ACC than non-musicians (*mean* = 69.37; *SD* = 10.99). No significant difference was observed between musicians and music learners.

However, no significant three-way interactions between musical elements, VFs, and groups were observed [*F*_(6, 186)_ = 0.28, *p* = 0.95], suggesting that musicians, music learners, and non-musicians did not show any different VFs effects among the four musical elements (pitch, harmony, rhythm, and articulation), in the ACC data.

### 3.2 Repeated measures analysis of variance (ANOVA) on the correct RT data

**Figure 8:**
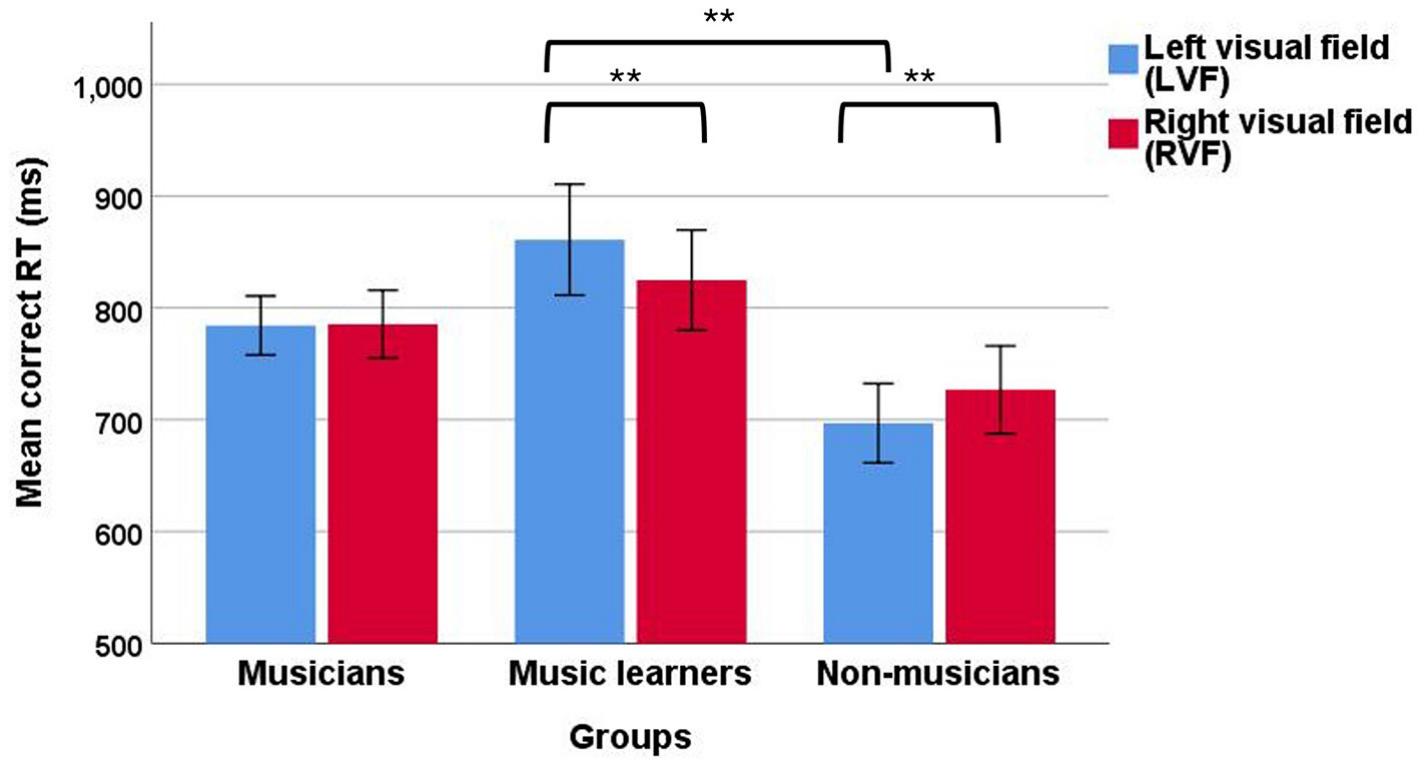
Mean correct RT of the divided visual field sequential matching tasks among musicians, music learners, and non-musicians across the two VFs (LVF and RVF; error bars show one standard error; **p < 0.01).

In the correct RT data, a significant two-way interaction between VFs and groups was observed, *F*_(2, 94)_ = 5.94, *p* = 0.004, ηp^2^ = 0.11, suggesting that there were different VF effects for musicians, music learners, and non-musicians during music reading, with a medium effect size. To further understand this two-way interaction, we first examined the data in separate groups. For musicians, no main effect of VF was observed [*F*_(1, 37)_ = 0.01, *p* = 0.94]. However, for music learners, a significant main effect of VF was observed, *F*_(1, 25)_ = 10.72, *p* = 0.003, ηp^2^ = 0.30, showing that music learners had a faster RT when musical elements were presented in the RVF (824.79 ms) than in the LVF (860.86 ms), with a large effect size. In contrast, for non-musicians, a significant main effect of VF was found, *F*_(1, 32)_ = 8.49, *p* = 0.006, ηp^2^ = 0.21, suggesting that non-musicians had a faster RT when musical elements were presented in the LVF (696.89 ms) than in the RVF (726.85 ms), with a large effect size (Figure 8). In addition, we examined the data from separate VFs among the three groups. In the LVF, a significant main effect of group was observed, with a large effect size, *F*_(2, 94)_ = 4.77, *p* = 0.011, ηp^2^ = 0.92. The *post-hoc* comparisons with Tukey HSD correction showed that non-musicians (*mean* = 696.89; *SD* = 203.59) had a faster correct RT in the LVF than music learners (*mean* = 860.86; *SD* = 252.93). In the RVF, no significant main effect of group was observed, *F*_(2, 94)_ = 1.62, *p* = 0.20, suggesting that the three groups did not differ in music reading in the RVF.

**Figure 9:**
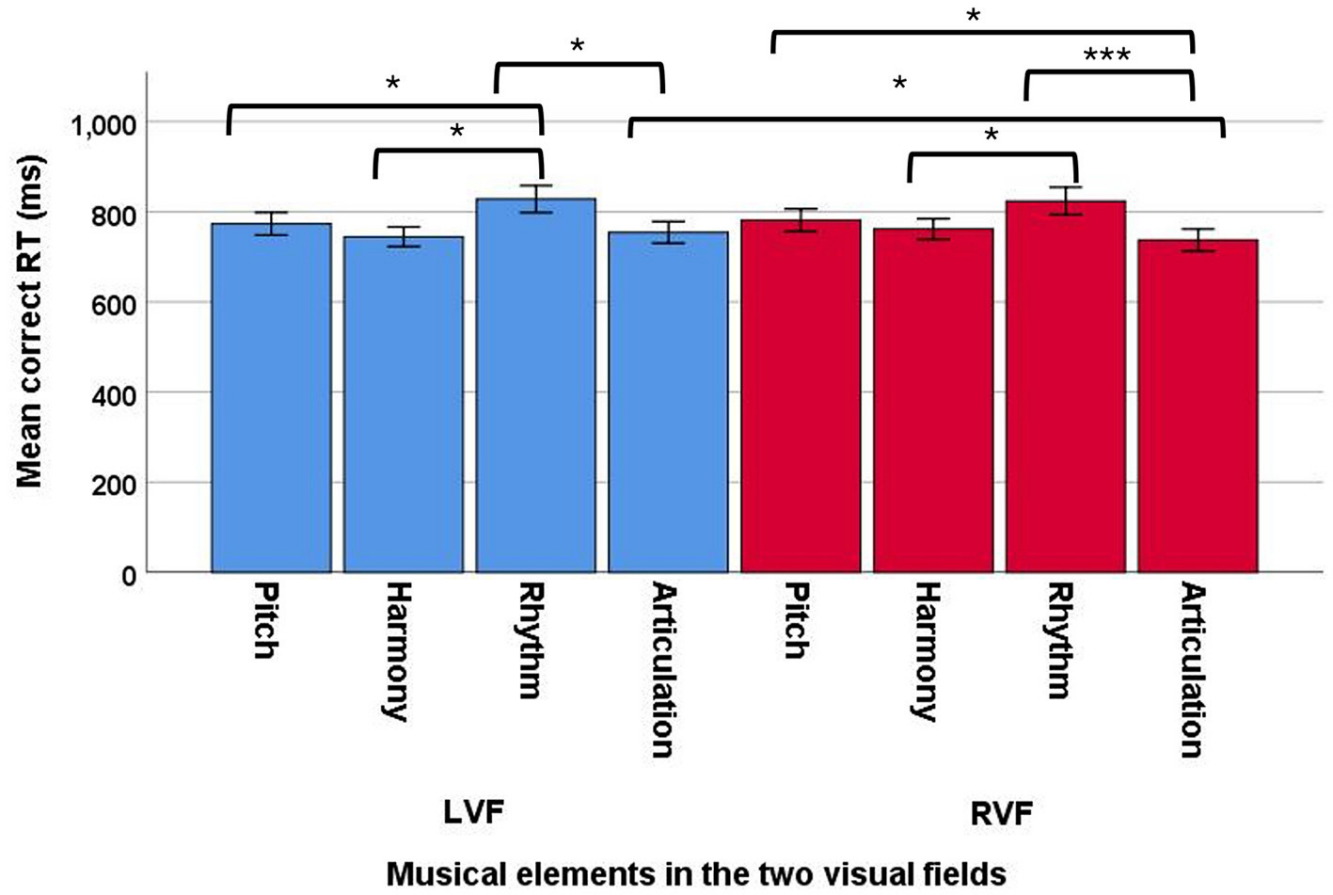
Mean correct RT of the four musical elements (pitch, harmony, rhythm, and articulation) examined in the divided visual field sequential matching tasks across the two VFs (LVF and RVF; error bars show one standard error; *p < 0.05, ***p < 0.001).

In addition, a significant two-way interaction between musical elements and VFs was observed, *F*_(3, 92)_ = 5.27, *p* = 0.001, ηp^2^ = 0.15, suggesting that various musical elements had different VF processing advantages, with a large effect size. To further understand this two-way interaction, we examined the data on different musical elements (Figure 9). No significant main effects of VF were observed for pitch [*F*_(1, 94)_ = 0.53, *p* = 0.47], harmony [*F*_(1, 94)_ = 2.38, *p* = 0.13], and rhythm [*F*_(1, 94)_ = 0.24, *p* = 0.63]. However, articulation showed a significant main effect of VFs, with a medium effect size [*F*_(1, 94)_ = 6.14, *p* = *0.0*15, ηp^2^ = 0.06]. These findings suggested that no particular processing advantages were found in either the LVF or RVF for pitch, harmony, and rhythm, while articulation showed a RVF (*mean* = 737.17; *SD* = 239.50) advantage over the LVF (*mean* = 754.20; *SD* = 236.81) in processing. In addition, we examined the data from separate VFs for the four musical elements. A significant main effect of musical elements was observed in the LVF, *F*_(3, 92)_ = 4.18, *p* = 0.008, ηp^2^ = 0.12, suggesting that the four musical elements resulted in different correct RTs in the LVF, with a medium effect size. The *post-hoc* comparisons showed that rhythm (*mean* = 836.51; *SE* = 28.94) had a significantly longer RT than pitch (*mean* = 777.15; *SE* = 25.50), harmony (*mean* = 751.43; *SE* = 21.36), and articulation (*mean* = 757.48; *SE* = 23.99). No other significant results were observed in the *post-hoc* comparisons. In addition, a significant main effect of musical elements was also observed in the RVF, *F*_(3, 92)_ = 4.57, *p* = 0.005, ηp^2^ = 0.13, suggesting that the four musical elements had different RTs in the RVF, with a medium effect size. The *post-hoc* comparisons showed that rhythm (*mean* = 829.80; *SE* = 30.26) had a significantly longer RT than harmony (*mean* = 769.59; *SE* = 23.28), and articulation (*mean* = 736.92; *SE* = 24.76), but not pitch (*mean* = 784.22; *SE* = 25.64). In addition, articulation also showed a significantly shorter RT than pitch and rhythm, but not harmony.

**Figure 10:**
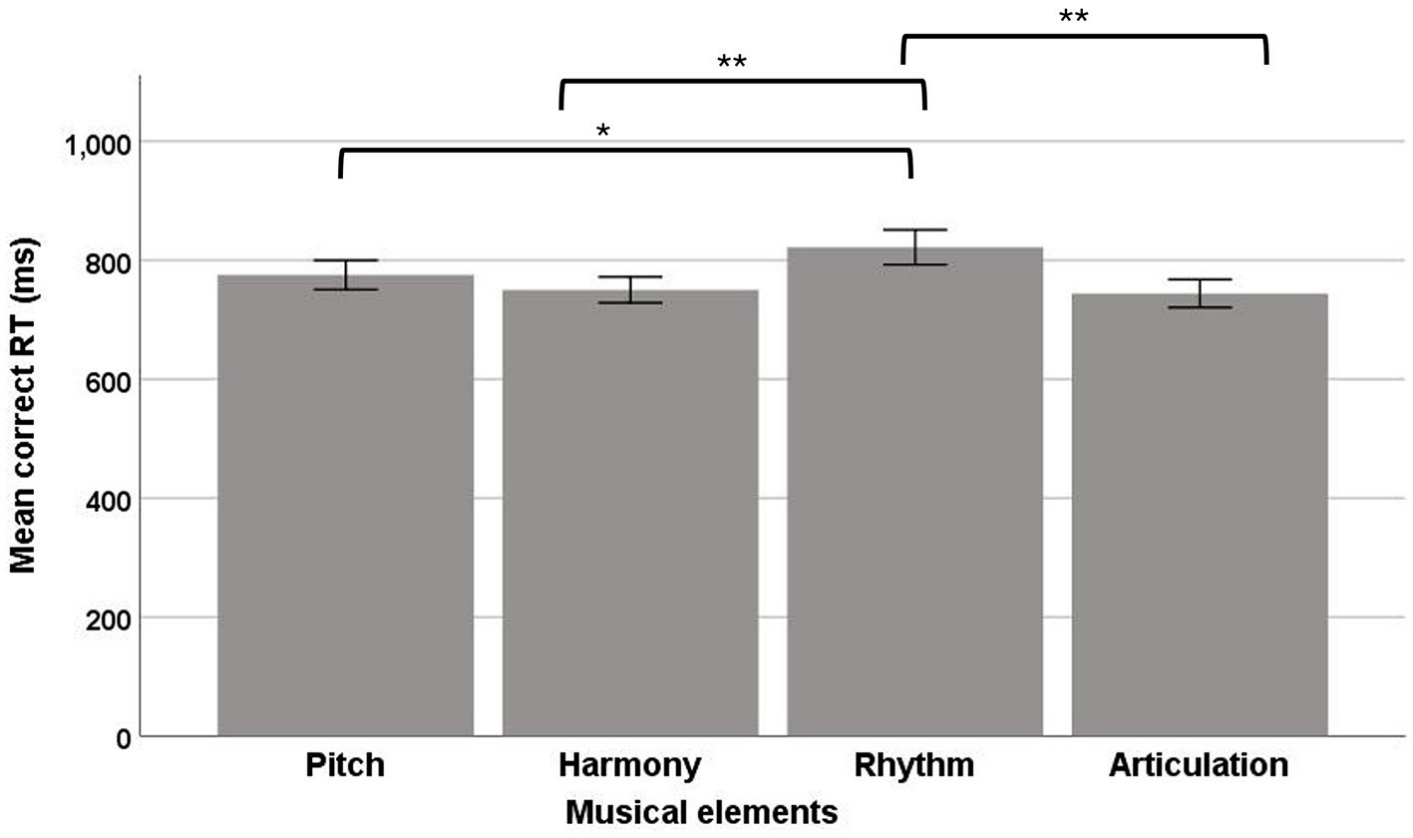
Mean correct RT of the four musical elements (pitch, harmony, rhythm, and articulation) examined in the divided visual field sequential matching tasks (error bars show one standard error; *p < 0.05, **p < 0.01).

In the correct RT data, a significant main effect of musical elements was observed, *F*_(3, 92)_ = 4.50, *p* = 0.005, ηp^2^ = 0.13, suggesting that the four musical elements had different RTs, with a medium effect size (Figure 10). The *post-hoc* comparisons showed that rhythm (*mean* = 833.16; *SE* = 28.81) had a significantly longer RT than pitch (*mean* = 780.69; *SE* = 25.11), harmony (*mean* = 758.27; *SE* = 21.89), and articulation (*mean* = 747.20; *SE* = 24.03).

A marginally significant main effect of groups was also observed [*F*_(2, 94)_ = 3.05, *p* = 0.052, ηp^2^ = 0.06], suggesting that musicians, music learners, and non-musicians had different RTs, with a medium effect size. The *post-hoc* comparisons showed that music learners (*mean* = 842.82; *SE* = 40.10) had a significantly longer RT than non-musicians (*mean* = 711.87; *SE* = 35.60). No significant difference was observed between musicians and music learners.

However, no significant three-way interactions between musical elements, VFs, and groups were observed [*F*_(6, 186)_ = 0.97, *p* = 0.45], suggesting that musicians, music learners, and non-musicians did not show any different VF effects among the four musical elements (pitch, harmony, rhythm, and articulation) in the correct RT data.

### 3.3 Laterality index in the ACC and correct RT data

The hemispheric lateralization effects among musicians, music learners, and non-musicians in music reading were further examined using the laterality index (LI). The laterality index ranges from −1 (indicating a right-lateralized processing advantage) to +1 (indicating a left-lateralized processing advantage; Seghier, 2008; Seghier et al., 2011). A repeated measures ANOVA was conducted with musical elements and groups as within-subject variables, while groups was the between-subject variables.

A laterality index analyses were conducted on the ACC data. In order to prevent possible misinterpretation of the laterality index based on the ACC data, a negative sign was inserted in the following formular to allow the same interpretation of the laterality index as in the RT data (i.e., −1 (indicating a right-lateralized processing advantage) to +1 (indicating a left-lateralized processing advantage; Seghier, 2008; Seghier et al., 2011).


$$\begin{array}{l} Laterality\;Index\;\left(LI\right)\;in\;ACC=-\frac{\left(LVF-RVF\right)}{\left(LVF+RVF\right)}\; \end{array}$$

In the laterality index based on the ACC data, no significant interaction and main effect of groups were found. A significant main effect of musical elements was found, *F*_(3, 92)_ = 2.96, *p* = 0.04, ηp^2^ = 0.09, suggesting that the four musical elements had different hemispheric lateralization with a medium effect size. The *post-hoc* comparisons showed that pitch (*mean* = 0.003; *SE* = 0.01) was more left lateralized than harmony (*mean* = −0.02; *SE* = 0.02), while articulation (*mean* = 0.002; *SE* = 0.01) was more left lateralized than harmony. No other significant comparisons were observed.


$$\begin{array}{l} Laterality\;Index\;\left(LI\right)\;in\;correct\;RT=\frac{\left(LVF-RVF\right)}{\left(LVF+RVF\right)}\; \end{array}$$

In the laterality index based on the correct RT data, no significant interaction was found. A significant main effect of groups was found, *F*_(2, 92)_ = 4.45, *p* = 0.01, ηp^2^ = 0.09, suggesting that musicians, music learners, and non-musicians differed in hemispheric lateralization of music reading, with a medium effect size. The *post-hoc* comparisons showed that music learners (*mean* = 0.019; *SD* = 0.03) had a more left-lateralized processing advantage than non-musicians (*mean* = −0.017; *SD* = 0.04), while non-musicians were more right lateralized than music learners in music reading. No significant comparisons were observed between musicians (*mean* = 0.003; *SD* = 0.57) and music learners or non-musicians in the laterality index.

In addition, a significant main effect of musical elements was found, *F*_(3, 92)_ = 4.87, *p* = 0.003, ηp^2^ = 0.14, suggesting that the four musical elements had different hemispheric lateralization, with a large effect size. The *post-hoc* comparisons showed that articulation (*mean* = 0.01; *SE* = 0.006) was significantly more left-lateralized than pitch (*mean* = −0.005; *SE* = 0.006), harmony (*mean* = −0.007; *SE* = 0.006), and rhythm (*mean* = 0.007; *SD* = 0.009). No other significant comparisons were observed.

### 3.4 Laterality index in the correct RT data and predefined thresholds (LI_*TH*_)

To further examine whether our findings of hemispheric lateralization observed in the correct RT data among the three groups fit into the predefined thresholds (LI_TH_) indicating left/bilateral/right dominance, a follow-up analysis was conducted. As shown in Seghier (2008), left hemispheric dominance is indicated by LI > LI_TH_, while right hemispheric dominance is indicated by LI < –LI_TH_. Bilateral dominance is indicated by |LI| ≤ LI_TH._ The threshold was set as 0.15 (see Baciu et al., 2005).

A one-sample *t*-test was used to examine whether the laterality index in the correct RT data among musicians, music learners, and non-musicians was significantly different from 0.15.

In the correct RT data, musicians' laterality index was significantly smaller than the predefined thresholds (0.15) (*mean* = 0.003, *SD* = 0.06), *t*_(37)_ = −16.00, *p* < 0.001, suggesting musicians' bilateral dominance in music reading. Nonetheless, music learners' laterality index was significantly smaller than the predefined thresholds (0.15) (*mean* = 0.019, *SD* = 0.03), *t*_(25)_ = −22.05, *p* < 0.001, suggesting music learners' bilateral dominance in music reading. Similarly, non-musicians' laterality index was significantly smaller than the predefined thresholds (0.15) (*mean* = |−0.017|, *SD* = 0.42), *t*_(32)_ = −22.94, *p* < 0.001, suggesting non-musicians' bilateral dominance in music reading when the laterality indices were compared with the predefined thresholds.

### 3.5 Correlations between other measures and the laterality Index in the correct RT data

To further explore how other measures correlated with the laterality index measures in the correct RT data, correlational analyses were conducted as follow.

**Figure 11:**
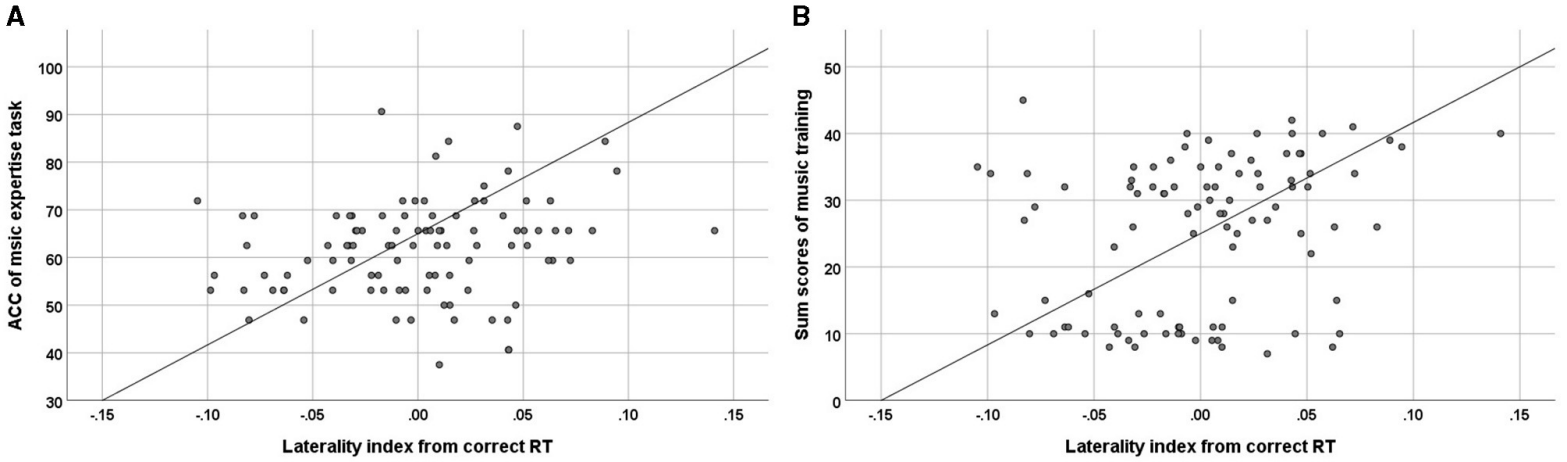
Scatterplots of the relationship between **(A)** the ACC of music expertise tasks and the laterality index measured in the correct RT [*r*_(97)_ = 0.207, *p* = 0.042) and **(B)** the sum scores of music training as measured in Gold-MSI (Müllensiefen et al., 2014) and the laterality index measured in the correct RT [*r*_(97)_ = 0.265, *p* = 0.009].

A significant positive correlation was found between the ACC of music expertise tasks and the laterality index measured in the correct RT data [*r*_(97)_ = 0.21, *p* = 0.04; Figure 11A left]. The higher the ACC in the music expertise tasks, the higher the observed laterality index value. This suggests that participants with a higher ACC in music expertise tasks tended to be more left lateralized in music reading. No other significant correlations were observed among ACC, or correct RT of music expertise tasks, and the laterality index in the correct RT and ACC data.

In addition, a significant positive correlation was found between the total scores of music training as measured in Gold-MSI (Müllensiefen et al., 2014) and the laterality index measured in the correct RT data [*r*_(97)_ = 0.27; *p* = 0.009; Figure 11B right]. No similar correlations were found between other subscales, including active engagement, perceptual abilities, singing abilities, emotions, and general musical sophistication.

### 3.6 Correlations between music training background and the ACC and correct RT data

To further explore how musicians' and music learners' music training background related to the ACC and the correct RT data, correlational analyses were conducted as follow.

Musicians' and music learners' onset of music learning was significantly correlated with overall ACC [*r*_(62)_ = −0.27, *p* = 0.037], overall correct RT [*r*_(62)_ = 0.35, *p* = 0.005], ACC in the LVF [*r*_(62)_ = −0.32, *p* = 0.011], correct RT in the LVF [*r*_(62)_ = 0.32, *p* = 0.011], and correct RT in the RVF [*r*_(38)_ = 0.36, *p* = 0.004]. These findings suggest that the earlier the onset of music learning, the higher the overall ACC, and the ACC in the LVF as shown in the divided visual field sequential matching task. Also, the earlier the onset of music learning, the faster the correct RT, and the correct RT in the LVF and RVF. No significant correlations were found between musicians' and music learners' onset of music learning and the ACC in the RVF and the laterality index in the ACC and RT data.

Similar correlational analyses between musicians' and music learners' years of experience in music playing and the ACC and correct RT data were conducted. Musicians' and music learners' years of experience in music playing was significantly correlated with overall ACC [*r*_(64)_ = 0.30, *p* = 0.015] and ACC in the RVF [*r*_(64)_ = 0.29, *p* = 0.021]. These findings suggested that the longer the years of experience in music playing, the higher the overall ACC and ACC in the RVF in the divided visual field sequential matching task. No significant correlations were found between musicians' and music learners' years of experience in music playing and overall correct RT, ACC in the LVF, correct RT in the LVF and RVF, and laterality index values in the ACC and RT data.

## 4 Discussion

This study examined how music expertise modulates the hemispheric lateralization of music reading among musicians, music learners, and non-musicians, and explore the effect of music training on brain plasticity. More specifically, this study investigated how music expertise modulates the hemispheric lateralization of music reading of pitch elements (e.g., pitch, harmony), temporal elements (e.g., rhythm), and expressive elements (e.g., articulation). Four main findings were shown as follow.

### 4.1 Music expertise differentially modulates the hemispheric lateralization of music reading

In this study, musicians, music learners, and non-musicians had shown different VF advantages in music reading. From the correct RT data of the divided visual field matching task, musicians had shown a bilateral representation in music reading based on the similar performance in the LVF and the RVF in the task. In addition, music learners tended to be more left-lateralized in music reading based their RVF/LH processing advantage over the LVF/RH in the task. In contrast, non-musicians tended to be more right-lateralized in music reading based on their LVF/RH processing advantage over RVF/LH in the task.

Some other findings were shown in the analysis of the laterality index (LI) comparing with the predefined threshold (LI_TH_; Baciu et al., 2005; Seghier, 2008). This classic measurement of hemispheric dominance showed that musicians had a bilateral dominance in music reading (indicated by |LI| ≤ LI_TH_). In addition, although music learners and non-musicians had also shown a bilateral dominance in music reading in music reading. When the laterality indices were compared with the predefined thresholds, some differential tendencies might still be able to observe based on the raw laterality indices across the three groups.

Additionally, music expertise was shown to be positively correlated with the laterality index (LI) in music reading. The finding suggested that the higher the music expertise, the greater the tendency to be more left-lateralized in music reading.

Our finding of music expertise differentially modulates the hemispheric lateralization of music reading was reflected in previous studies. Musicians' bilateral processing advantage in music reading has been shown in neuroimaging studies. For example, an early study suggested a bilateral activation of the extrastriate visual areas during music reading (Sergent et al., 1992). Additionally, in an fMRI study, Mongelli et al. (2017) has shown that musicians had bilateral left-predominant cortical activations in the lateral and mesial prefrontal and rolandic cortex, IPS, temporal and occipital lobe, and right-predominant cerebellum during music reading. It is important to note that the music expertise-related network was found to be predominated in the left hemisphere (Mongelli et al., 2017). The left-lateralized perisylvian regions, left intraparietal cortex, and bilateral ventral temporal cortex were regions specified for music expertise. These findings provided insights to explain musicians' bilateral processing advantage in music reading. In addition, musicians had active representations of musical elements that are readily engaged in music reading (Sloboda, 1974). This idea is further supported by Patston et al. (2007), in which both groups performed similarly when the dots were presented to the left of the line, musicians outperformed non-musicians when the dots were presented to the right of the line. This finding has further suggested that musicians had a more bilateral attention capacity as compared with non-musicians in visual processing. Thus, musicians could process music more flexibly in both hemispheres.

Another speculation to explain musicians' bilateral processing advantage in music reading could be related to notation-evoked sound imagery (i.e., notational audiation, in which professional music players are able to image the sound of music from musical notations; Wolf et al., 2018). The sound imagery may potentially involve various cognitive processes with different hemispheric lateralization effects, such as music auditory processing, that tends to be more right-lateralized (Tervaniemi and Hugdahl, 2003), and auditory imagery, that tends to be more left-lateralized (Prete et al., 2016). On the other hand, individual differences among musicians may further complicate the investigation. Musicians who were absolute pitch possessors have shown stronger activations in the right-sided perisylvian network when compared to musicians who were relative pitch possessors (Burkhard et al., 2019). It remains unclear how these cognitive processes interact with one another during music reading among musicians. More studies are needed to figure out the underlying cognitive processes and neural correlates of notational audiation, and whether and how it relates to musicians' hemispheric lateralization in music reading.

Concerning the findings of music learners' left-lateralized processing advantage in music reading, this finding was supported by an fMRI study conducted by Muayqil et al. (2015). Participants with some basic music literacy has shown different activations in the left inferior frontal gyrus between musical notation and its non-symbolic equivalent. In addition, among the few studies examining music learners' hemispheric lateralization in music reading, Stewart et al. (2003) has shown that participants who learned piano and score reading for 15 weeks showed a bilateral activation in the superior parietal cortex in a sight-reading task. Some specific training effects were also observed in the left supramarginal gyrus, left inferior frontal sulcus, and right frontal pole. Both studies further highlighted the possibility that music learners had a left-lateralized processing tendency in music reading.

Additionally, music learners' left-lateralized processing advantage in music reading may be explained further by the use of analytical processing strategy in the divided visual field sequential matching task. Music learners may focus more on details on compare between the two target stimuli given their basic music literacy. The analytical processing was shown to be left-lateralized (Bever and Chiarello, 1974), which might further support our findings.

Our findings of non-musicians' right-lateralized processing advantage in music reading are also consistent with previous findings in neuroimaging studies (e.g., Proverbio et al., 2013; Pantaleo et al., 2024). As novices, musical notations did not carry specific meanings to non-musicians, and thus they might employ different processing strategies to finish the divided visual field sequential matching task. One possibility is that non-musicians may employ different visuo-spatial processing strategies (e.g., comparing the location of notes in pitch/harmony matching; differentiating visual features when matching articulations), which tended to be right-lateralized (Bever and Chiarello, 1974; Patston et al., 2006).

### 4.2 Similar hemispheric lateralization effects were observed for pitch, temporal, and expressive elements across different music expertise

To further explore how different musical elements were processed among different music expertise, this study examined whether musicians, music learners, and non-musicians differ in hemispheric lateralization in pitch elements (e.g., pitch, harmony), temporal elements (e.g., rhythm), and expressive elements (e.g., articulation), respectively. No significant interactions were observed among visual fields, musical elements, and groups, indicating that musicians, music learners, and non-musicians did not show different visual field effects in any individual musical elements respectively.

This finding is also consistent with other previous studies showing that no hemispheric lateralization effects were observed in the musical features examined in the auditory domain, including pitch, chord, rhythm, and timbre, among musicians and non-musicians (Ono et al., 2011). Our study may also be among the first few to show that no significant hemispheric lateralization differences were shown in individual musical elements among participants with different music expertise. A few speculations may explain this finding. First, it may suggest that the processing of pitch, harmony, rhythm, and articulation involve resources from the two hemispheres, while musicians, music learners, and non-musicians may not differ much in the cognitive processes when only one particular musical element is examined. Or alternatively, it may suggest that the processing of pitch, harmony, rhythm, and articulation might involve different cognitive processes among musicians, music learners, and non-musicians when only one particular musical element was examined, but then these cognitive processes might share similar lateralization effects as observed.

The qualitative difference between music reading in general and music reading focusing on a musical element remains uncertain. To speculate, it may be that further suggest hemispheric lateralization is sensitive to music reading in general, but not to music reading focusing on a particular musical element. More research is needed to investigate this matter.

### 4.3 Both hemispheres are involved in the reading process of pitch, temporal, and expressive elements

Our findings showed that pitch, harmony, and rhythm tended to be bilateral in processing, while articulation remained either bilateral or left-lateralized in processing as shown in different analyses.

The bilateral processing advantage of pitch tends to be similar to previous studies showing no hemispheric lateralization effect in behavioral studies (e.g., Li and Hsiao, 2018) or bilateral processing in neuroimaging studies (Lu et al., 2019; Proverbio et al., 2024). This may potentially reflect the right-lateralized global processing and left-lateralized local processing (Fink et al., 1997) involved in pitch reading. Music readers needed to attend to the five-line staff as global information, as well as the specific note locations across the five lines and four spaces as local information.

A similar bilateral processing advantage was also observed in harmony as shown in behavioral studies (e.g., Li and Hsiao, 2018). This again may reflect the right-lateralized global processing of staff and left-lateralized local processing (Fink et al., 1997) of specific note locations involved in harmony reading. Some previous studies had shown that chord reading was found to be left-lateralized in musicians or amateur musicians (Segalowitz et al., 1979; Salis, 1980). However, this may possibly due to the task nature of the studies, in which participants needed to play the harmony on a keyboard after reading chords on scores. Motor planning and control was found to be left-lateralized (Mutha et al., 2012). Since our divided visual field sequential matching task did not involve music playing, and thus no left-lateralized processing advantage was observed in chords.

Furthermore, bilateral processing advantages were also observed in rhythm and articulation. These findings were among one of the few studies examining the hemispheric lateralization effects of reading rhythmic and articulatory patterns. These could be explained by the right-lateralized global processing (Fink et al., 1997) of the overall rhythmic and articulatory patterns, and left-lateralized local processing (Fink et al., 1997) of specific rhythmic and expressive markings. The findings from Salis (1980) examining the hemispheric lateralization of dot enumeration (i.e., perceiving spatial relations and groupings among dots) among musicians may also provide some insight to explain the role of the right hemisphere perceiving spatial relations and groupings among items, as in rhythmic and articulatory processing.

Among the few studies examining hemispheric lateralization of different musical elements, this study showed that the processing of pitch, harmony, rhythm, and articulation involved resources from the two hemispheres. The similar results of our study and previous studies (e.g., Ono et al., 2011) indicate that it remains uncertain whether the visual and auditory processing of musical elements may have some overlaps in the cognitive processing and neurocognitive bases.

### 4.4 Music training specifically relates to hemispheric lateralization of music reading

Music training was the only subscale in Gold-MSI (Müllensiefen et al., 2014) that showed a positive correlation with the laterality index (LI) in music reading. This finding highlights the uniqueness of music training on hemispheric lateralization among other measurements of musicality in Gold-MSI, including active engagement, perceptual abilities, singing abilities, emotions, and general musical sophistication. Nonetheless, we remain conscious about the interpretation regarding correlational nature of this finding.

## 5 Conclusion

To conclude, this study explored how music expertise modulates the hemispheric lateralization in music reading among musicians, music learners, and non-musicians. Musicians had demonstrated a bilateral processing advantage in music reading. Music learners tended to be more left-lateralized in music reading, while non-musicians tended to be more right-lateralized in music reading. Music expertise correlates with the laterality index in music reading, suggesting the higher music expertise, the greater the tendency to be more left-lateralized in music reading. Musicians, music learners, and non-musicians did not show different visual field effects in any individual musical elements, including pitch, harmony, rhythm, and articulation respectively. The uniqueness of music training on hemispheric lateralization in music reading has also been highlighted.

In short, this study further suggests the effect of music training on brain plasticity along the music learning trajectory. It also highlights the possibilities that the bilateral or left hemispheric lateralization may serve as an expertise marker for musical reading.

## Data availability statement

The datasets presented in this study can be found in online repositories. The names of the repository/repositories and accession number(s) can be found in the article/supplementary material.

## Ethics statement

The studies involving humans were approved by Research Ethics Committee (REC), Hong Kong Metropolitan University. The studies were conducted in accordance with the local legislation and institutional requirements. The participants provided their written informed consent to participate in this study.

## Author contributions

SL: Conceptualization, Data curation, Formal analysis, Funding acquisition, Investigation, Methodology, Project administration, Resources, Software, Supervision, Validation, Visualization, Writing – original draft, Writing – review & editing.
